# Potentiometric Detection of Calcium Ions Using an Organic Electrochemical Transistor

**DOI:** 10.1021/acsomega.5c03720

**Published:** 2025-07-18

**Authors:** Danilo Arcangeli, Federica Mariani, Isacco Gualandi, Manuel Ragnucci, Francesco Decataldo, Filippo Bonafè, Domenica Tonelli, Beatrice Fraboni, Erika Scavetta

**Affiliations:** † Organic Bioelectronics Laboratory, Biological and Environmental Science and Engineering Division (BESE), 127355 King Abdullah University of Science and Technology (KAUST), Thuwal 23955-6900, Saudi Arabia; ‡ Department of Industrial Chemistry “Toso Montanari”, 9296 University of Bologna, Via Piero Gobetti 85, Bologna 40129, Italy; § Department of Physics and Astronomy “Augusto Righi”, University of Bologna, Viale Berti Pichat 6/2, Bologna 40127, Italy

## Abstract

Excreted biological fluids represent a matrix rich in useful biomarkers that can be correlated with a vast number of medical conditions and physiological processes, for both diagnostic and therapeutic purposes. Among the plethora of medically relevant analytes, the calcium ion is a target of prime interest, as it is present in many biofluids such as saliva, sweat, and wound exudate. Its concentration can be associated with a variety of afflictions, such as osteoporosis, periodontal diseases, kidney stone formation, parathyroid gland activity, and impaired wound healing. In this work, we present a novel device architecture for the potentiostatic, reference electrode-free detection of calcium, renovating the original organic electrochemical transistor (OECT) model by Wrighton. The sensing event takes place on the gate, functionalized with a Ca^2+^-selective membrane, whose potentiometric shift is used to modulate the current flowing through a poly­(3,4-ethylenedioxythiophene) perchlorate (PEDOT:ClO_4_) interdigitated channel. Calcium sensing was performed in buffer solutions, reporting a linear range between 0.002 and 19 mM. The sensor proved to cover the biologically relevant range for calcium ion concentrations in complex media, using synthetic wound exudate as a model biomatrix, and it reported excellent selectivity under exposure to high concentrations of interfering species.

## Graphical abstract

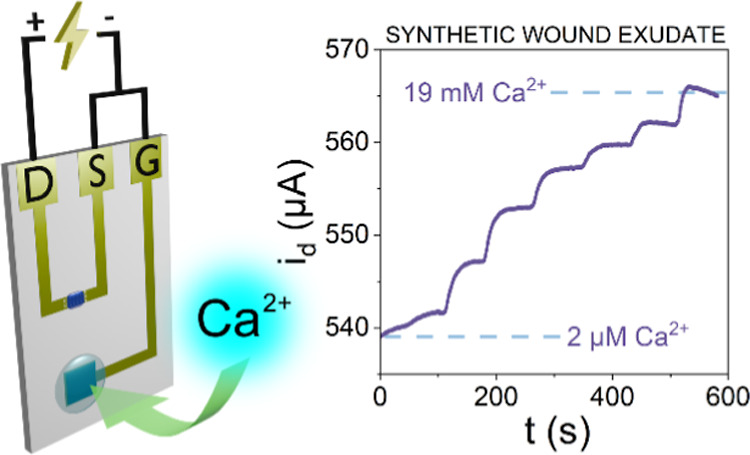

## Introduction

Over the years, ion detection has proven to be a field of research full of medically relevant applications: quantifying the concentration of a specific ion in a certain biological fluid can be crucial in aiding diagnostics for a broad range of illnesses, tailoring their relative therapies, or assessing the global health conditions of an individual. The insight provided by the quantification of ion concentration is especially attractive when contextualized in a point-of-care approach: the analysis must be performed in situ with quick, user-friendly, and noninvasive techniques, implying the sampling of saliva, urine, sweat, or wound exudate (alternative to blood). Among the different ions that can be detected, calcium is particularly interesting, as research studies have proven its association with many medical conditions, depending on the biofluid where it is determined. In saliva, calcium quantification has been used to screen for osteoporosis^1^ and periodontal diseases,^2^ while in urine, it has shown potential in diagnosing parathyroid gland dysfunctions^3^ and kidney stone formation.^4^ In other excreted body fluids, such as wound exudate, calcium detection could be used to assess the wound health status^5^ as a higher calcium concentration was found to be associated with an increased healing rate and better outcomes in both acute and chronic wounds.^6^ Lastly, when focusing on sweat, it has been observed that calcium presence affects the sodium content of the biofluid, specifically promoting sodium ion reabsorption when present in low concentrations.^7,8^ For clarity, Table 1 summarizes the approximate average ranges of the calcium ion concentration in different biological fluids.

**Table 1:** Summary of Average Biological Ranges for Calcium in Different Biological Fluids

fluid	calcium concentration	references
plasma	2.2–2.6 mM (total)	Foley and Boccuzzi^9^
saliva	0.88–2.05 mM (free)	Rehak et al.^10^
urine	1.7–5.0 mM (total)	Foley and Boccuzzi^9^
sweat	1–10 mM (total)	Sato et al.^11^
wound exudate	2.15–2.5 mM (total)	Trengove et al.^12^

Many options are present in the modern scientific literature for point-of-care (PoC) calcium sensing, featuring devices based on a variety of principles, the most common of which is the potentiometric method, consisting of an ion-selective electrode (ISE) coupled to a reference electrode (RE). Specifically, solid-contact ISEs (SC-ISEs) are among the most used for PoC applications, as pioneered in the works of Bobacka et al.^13,14^ Starting from the original coated-wire design, which removed altogether the necessity for a filling solution, crucial in standard bulk ISEs, allowing for a significant miniaturization of the electrode, the nature of the solid contact has experienced significant evolution over the last few decades. Nowadays, one of the most reported architectures consists of an ion-selective membrane (ISM) deposited on a high-capacitance ion-to-electron transducer, which enables charge transfer at the interface between the ion-conducting membrane and the electron-conducting metal electrode, thus improving the robustness of the sensing mechanism. As such, focusing on calcium detection, the most recent examples of SC-ISEs in the literature involve PoC-friendly substrates such as paper, textile materials, or plastic films, employing solid contact materials such as conductive polymers or carbon-based compounds functionalized with a calcium-selective membrane.^15–19^ For such devices, the instrumental response is ruled by the Nernst equation, which can be described as in eq 1 for positively charged, bivalent ion selective electrodes, where “*a*_*i*_” is the activity of the ion of interest, “*k*” is the electrode constant, and 0.02958 (expressed in volts) is a constant that resumes the charge of the ion “*z*”, Faraday’s constant “*F*” (C/mol), the ideal gas constant “*R*” (J mol^–1^ K^–1^), and temperature “*T*” (K).

1 $$E=k+0.02958\;\log\left[a_{i}\right]$$

Equation 1 highlights that for ion-selective systems based on pure potentiometric detection, the maximum sensitivity that can be achieved is thermodynamically limited and equal to 29.58 mV dec^–1^ at *T* = 25 °C. Consequently, in a potentiometric application, the sensitivity decreases as the ion charge increases, and therefore, the relative error increases. Besides these limitations, PoC potentiometric sensors are also affected by another issue, which is the miniaturization of the reference electrode. As such, it is often more appropriate to refer to them as pseudo-reference electrodes since these systems usually consist of bare Ag/AgCl composites, lacking a filling solution and a porous frit. This exposes the reference electrode to the sampling environment, which, in biological matrixes, can cause fouling and interferences, for example, a change in the chloride ion concentration, leading to unwanted electrochemical potential shifts and drifts.^20^ Moreover, unshielded Ag/AgCl composites are susceptible to Ag^+^ ion leaching, thus representing an unnecessary source of a heavy metal cation possessing cytotoxic activity, potentially interfering with biological processes.^21–23^ The necessity of a pseudoreference electrode can be removed, as described in the work by Keene et al.^24^ where organic electrochemical transistors (OECTs) consisting of a channel made of poly­(3,4-ethylenedioxythiophene)/polystyrenesulfonate (PEDOT:PSS) and a Ag/AgCl gate electrode were functionalized with a calcium and ammonium ion selective PVC-based membrane covering the whole device, using a styrene–ethylene–butylene–styrene (SEBS) elastomer as a substrate. In such a configuration, contrary to potentiometric systems, the Ag/AgCl composite serves as a high-capacitance material (i.e., behaving as a nonpolarizable electrode) to maximize the gating effect on the PEDOT:PSS channel, i.e., altering its redox state. The presence of the membrane on both the channel and gate electrode only allows the ions of interest to participate in the doping–dedoping reactions involving the channel, while preventing Ag^+^ from leaking. Specifically, a pulsed potentiostatic technique was chosen to detect and quantify calcium and ammonium ions, spanning several orders of magnitude in concentration. A high sensitivity was achieved along with signal reversibility and good selectivity over other ions such as sodium and potassium. In other works,^25,26^ the ISM was applied on the channel of an OECT, respectively, using indium–gallium–zinc oxide and a silver wire as gate electrodes. In both cases, ion-sensing, including calcium, was achieved by potentiodynamic techniques using the drain current as the output signal. A similar concept is implemented in a paper-based OECT for the detection of potassium ions, highlighting the versatile use of ion-sensing transistors on cheap and commonly available substrates.^27^ In such OECT configurations, the driving force behind the recorded signal used for calcium sensing can be ascribed to a change in the ionic conductivity of the membrane, which is proportional to the target ion concentration rather than a potentiometric shift obtained via spontaneous electrochemical phenomena, differing significantly from classic potentiometric approaches in common ISEs. In that way, the effect obtained is comparable to a shift in the effective *V*_g_ needed to change the redox state of the channel, which does not fully take advantage of the electrochemical potential shift generated across an ion-selective membrane. Moreover, using OECTs involving Ag-based gate electrodes can lead to a lower selectivity, as the electrochemical potential of silver composites is strongly affected by the concentration of certain anions (most notably chloride), which might be subject to significant changes, especially in biological applications. Another relevant example of ion-sensing OECT is reported in the work of Han et al.,^28^ where it is argued that a channel coated with an ISM responds to ion concentration via the potential difference generated at the membrane–analyte interface, in addition to demonstrating device operation at *V*_gs_ = 0. In order to address these issues and to provide alternative approaches to ion sensing, we are presenting in this work a novel device configuration, inspired by one of the first OECT architectures presented by Mark Wrighton’s group^29^ featuring a solid-contact ion-selective PEDOT:PSS/Au gate electrode externally short-circuited to the source terminal of a PEDOT:ClO_4_ interdigitated channel, creating a potentiometrically controlled OECT for the potentiostatic detection of calcium ions. This new configuration permits the decoupling of the interactions between the ions and the polymeric channel from Nernstian equilibria involving the ISM and allows for an in-depth study of the electrochemical phenomena as the basis of the desired sensing, providing higher control in terms of selectivity. This novel device, termed “pseudo-Wrighton”, was tested for calcium sensing in buffer solutions and in synthetic wound exudate, displaying high selectivity even when exposed to large amounts of interfering ions and organic molecules. The characterizations performed, encompassing a thorough material study and a decoupling of the electrochemical components of the OECT with respect to the analyte, proved that the potentiometric shift of the ion-selective gate electrode is the driving force in changing the redox state of the channel, akin to what described in the work^30^ by Gualandi et al., where the potentiostatic determination of different anions was achieved by using Ag/AgX composites on the gate electrode as potentiometric transducers on a PEDOT:PSS channel to obtain drain current modulation. Globally, this device represents an interesting alternative to classic potentiometric and OECT-based ion sensing, incorporating functionalities and principles from both strategies. The result is an extremely simple and versatile sensor, devoid of any silver-based interface and requiring bare-bones readout electronics, whose response could potentially be tailored to detect any ion using the appropriate ionophore. In addition, particular attention was addressed to device selectivity through the development of a quantitative method based on the adaptation of the Nikolsky–Eisenman fixed interference method to the OECT.

## Experimental Section

### Chemicals and Buffers

Clevios PH1000 suspension (PEDOT:PSS) was purchased from Heraeus. Sodium hydroxide, sodium chloride, potassium chloride, potassium nitrate (containing 7.9 mg/kg Ca impurities, equal to 2 μM Ca^2+^ for a 0.1 M KNO_3_ solution), urea, d-(+)-glucose, sodium l-lactate, l-lactic acid, albumin, alkaline phosphatase (ALP), sodium polystyrenesulfonate (NaPSS), lithium perchlorate, ethylenedioxythiophene (EDOT), barium chloride, *N*,*N*-dicyclohexyl-*N*′,*N*′-dioctadecyl-3-oxapentanediamide (ETH5234, Calcium Ionophore IV), *ortho*-nitrophenyloctyl ether (*o*-NPOE), anhydrous inhibitor-free tetrahydrofuran (THF), ethylene glycol (EG), dodecyl benzenesulfonic acid (DBSA), 3-glycidoxypropyl trimethoxysilane (GOPS), and agarose were purchased from Merck. High-molecular-weight polyvinyl chloride (HMW-PVC) was obtained from Fluka. Magnesium nitrate hexahydrate and potassium tetrakis­(4-chlorophenyl)­borate were acquired from Alfa Aesar. Strontium chloride was purchased from Carlo Erba. The conductive silver paste was obtained from RS Components. All chemicals used were reagent-grade or higher. Simulated wound exudate (SWE)^31^ was prepared by mixing 0.005 M KCl, 0.009 M urea, 0.002 M d-(+)-glucose, 0.009 M l-lactic acid, 22 g/L albumin, and 84 U/L ALP in a solution of 0.1 M NaH_2_PO_4_ adjusted to pH 7.00 with 1 M NaOH as a pH buffer and source of Na^+^ ions. 4000 grit silicon carbide sanding paper discs were obtained from Remet, while 0.05 μm alumina powder was acquired from Buehler. A Microposit S1818 positive photoresist, Microposit MF-319 developer, mr-DWL 5 negative photoresist, and mr-DWL 5 negative photoresist were purchased from Micro Resist Technology.

### Apparatus

Bulk gold and glassy carbon (GC) electrodes were acquired from BASi. OECT microfabrication through photolithography was performed using an ML3Microwriter from Durham Magneto Optics. Potential-controlled and 2-terminal-based measurements were conducted using a CH Instruments 900B bipotentiostat. Simultaneous potential-controlled and open-circuit potential (OCP) measurements were conducted by coupling a Keysight B2902A Precision Source/Measure Unit (for two-channel *i*–*t* measurements) to a CH Instrument 900B potentiostat (for the OCP-*t* measurements). Electrode potentials were applied against an aqueous saturated calomel reference electrode (SCE) while using a Pt wire as a counter electrode (CE) when needed. All solution-based pH measurements were conducted by using a combined glass electrode (Amel 411/CGG/12) connected to a pH meter (Amel Instruments 338). All measurements were conducted at room temperature and pressure, unless specified otherwise.

### Ion-Selective Electrode Preparation

The Au (diameter = 1.5 mm) and glassy carbon (diameter = 3.0 mm) electrodes were polished on 4000 grit silicon carbide sanding paper discs and successively on a suspension of water and 0.05 μm alumina powder. After being rinsed with water, they were sonicated in acetone for 5 min. Once dry, PEDOT:PSS was electrochemically polymerized on their surfaces in a 3-electrode cell using a saturated calomel as a reference electrode and a platinum gauze as a counter electrode in a 0.1 M KNO_3_ solution containing 10 mM EDOT and 0.1 mM NaPSS. The electrode potential was then scanned from 0 to 1.1 V vs SCE for 10 cycles at 100 mV s^–1^. Once functionalized with the conductive polymer, the electrodes were rinsed with deionized water and left to precondition in 19 mM CaCl_2_ overnight, rinsed again, and dried at room temperature. The ISM cocktail was prepared according to the following procedure adapted from the literature:^32,33^ a 25 mg mL^–1^ solution of potassium tetrakis­(4-chlorophenyl)­borate and a 20 mg mL^–1^ solution of ETH5234 in anhydrous inhibitor-free THF were prepared. Another solution was made by mixing 2.9 mg of HMW-PVC and 9 μL of *o*-NPOE in 170 μL of anhydrous inhibitor-free THF, which was then left to sonicate until homogeneous. To the latter solution were then added 2 μL of the potassium tetrakis­(4-chlorophenyl)­borate solution and 30 μL of the ETH5234 solution, constituting the final calcium cocktail solution. On the previously prepared Au and glassy carbon electrode surfaces, 5 μL of calcium cocktail was drop-cast using a micropipet and left to dry at room temperature. Afterward, the electrodes were treated in a temperature-controlled oven at 72 °C for 15 min. The ion-selective electrodes were then subjected to a forced conditioning process in 0.1 M CaCl_2_ in a 3-electrode cell for 400 s under an electrode potential of −0.1 V vs SCE. Successively, they were rinsed and left to passively condition for a minimum of 24 h in a 10 mM CaCl_2_ solution.

### Bulk and Gate Electrode Ca^2+^ ISE Potentiometric Measurements

The Ca^2+^ ISEs produced on bulk and gate electrodes were characterized by a potentiometric calibration. The electrodes were immersed in 20 mL of 0.1 M KNO_3_ under stirring while performing addition of CaCl_2_ from a standard solution. In the case of ISEs produced on glass-based devices, only the gate electrode was connected to the potentiostat and submerged in the electrolyte. The open-circuit electrochemical potential was measured over time by using a saturated calomel electrode as a reference electrode.

### Wrighton OECTs Production

Glass substrates were cleaned by sonication in acetone/isopropyl alcohol/distilled water baths. After the dehydration step (10 min at 110 °C), the Microposit S1818 positive photoresist (from Micro Resist Technology) was spin-coated (4000 rpm for 60 s) and annealed at 110 °C for 1 min. Metallic contacts were patterned through direct laser lithography using an ML3Microwriter (from Durham Magneto Optics). The photoresist was developed with a Microposit MF-319 developer. Then, 7 nm of chromium and 30 nm of gold were deposited by thermal evaporation. Samples were immersed in acetone for 4 h for photoresist liftoff. Metallic contacts were encapsulated by using an mr-DWL 5 negative photoresist (from Micro Resist Technology). The resin was spin-coated at 3000 rpm for 30 s and annealed at 100 °C for 2 min. After laser exposure, samples were baked at 100 °C for 2 min, relaxed for 1 h at room temperature, and developed with an mr-Dev 600 developer (Micro Resist Technology). A final oxygen plasma descum of photoresist residuals (120 W for 4 min) was performed, and then the negative resist was baked at 120 °C for 30 min. A double layer of S1818 was deposited to pattern the PEDOT:PSS microstructures (the OECT channel). After development, substrates were treated with air plasma (15 W for 2 min), and the PEDOT:PSS solution (94% PEDOT:PSS (Heraeus, Clevios PH1000), 5% ethylene glycol (EG) (Sigma-Aldrich), 1% 3-glycidoxypropyltrimethoxysilane (GOPS), and 0.25% 4-dodecylbenzenesulfonicacid (DBSA)) was spin-coated at 3000 rpm for 10 s. The resulting film thickness was (100 ± 10) nm. The samples were subsequently annealed at 120 °C for 1 h, and S1818 was finally lifted off after 4 h in isopropanol. The geometry of the Wrighton-OECT is reported in Figure S1, featuring a spin-coated PEDOT:PSS channel and gate electrode. The gate electrode was then used as the substrate to produce the Ca^2+^ ISE following the procedure described in the previous paragraph.

### Pseudo-Wrighton OECTs Production

Glass substrates were cleaned by sonication in water and soap (10%)/acetone/isopropanol/distilled water baths. After the dehydration step (10 min at 110 °C), the Microposit S1818 positive photoresist (from Micro Resist Technology) was spin-coated (4000 rpm for 60 s) and annealed at 110 °C for 1 min. Metallic contacts were patterned through direct laser lithography using the ML3Microwriter (from Durham Magneto Optics). The photoresist was developed with a Microposit MF-319 developer. Then, 7 nm of chromium and 30 nm of gold were deposited by thermal evaporation. Samples were immersed in acetone for 4 h for photoresist liftoff. Metallic contacts were encapsulated using an mr-DWL 5 negative photoresist (from Micro Resist Technology). The resin was spin-coated at 3000 rpm for 30 s and annealed at 100 °C for 2 min. After laser exposure, samples were baked at 100 °C for 2 min, relaxed for 1 h at room temperature, and developed with mr-Dev 600 developer (Micro Resist Technology). A final oxygen plasma descum of photoresist residuals (120 W for 4 min) was performed, and then, the negative resist was baked at 120 °C for 30 min. The geometry of pseudo-Wrighton-OECT is reported in Figure 1a. The interdigitated channel was closed by electropolymerizing PEDOT:ClO_4_ from a solution containing 0.1 M LiClO_4_ and 10 mM EDOT. In all the electropolymerizations performed, a 3-electrode cell setup was used, featuring a saturated calomel as RE and a Pt gauze as CE. The WE potential was scanned from 0 to 1.1 V vs SCE at 100 mV/s for 5 cycles. The electrodeposition process was repeated 3 times to promote the full electrical contact among the interdigitated channel fingers, each time connecting, respectively, the source terminal, the drain terminal, and then both. The gate electrode was functionalized with PEDOT:PSS via electrochemical polymerization in a 3-electrode cell using a saturated calomel as a reference electrode and a platinum gauze as a counter electrode in a 0.1 M KNO_3_ solution containing 10 mM EDOT and 0.1 mM NaPSS. The electrode potential was then scanned from 0 to 1.1 V vs SCE for 10 cycles at 100 mV s^–1^. Once functionalized with the conductive polymer, the electrodes were rinsed with deionized water and left to precondition in 19 mM CaCl_2_ overnight, rinsed again, and dried at room temperature. Subsequently, 5 μL of calcium cocktail was drop-cast using a micropipette and left to dry at room temperature. Afterward, the electrodes were treated in a temperature-controlled oven at 72 °C for 15 min. The ion-selective gate electrodes were then subjected to a forced conditioning process in 0.1 M CaCl_2_ in a 3-electrode cell for 400 s under an electrode potential of −0.1 V vs SCE. Afterward, they were rinsed and left to passively condition for a minimum of 24 h in a 10 mM CaCl_2_ solution.

**Figure 1:**
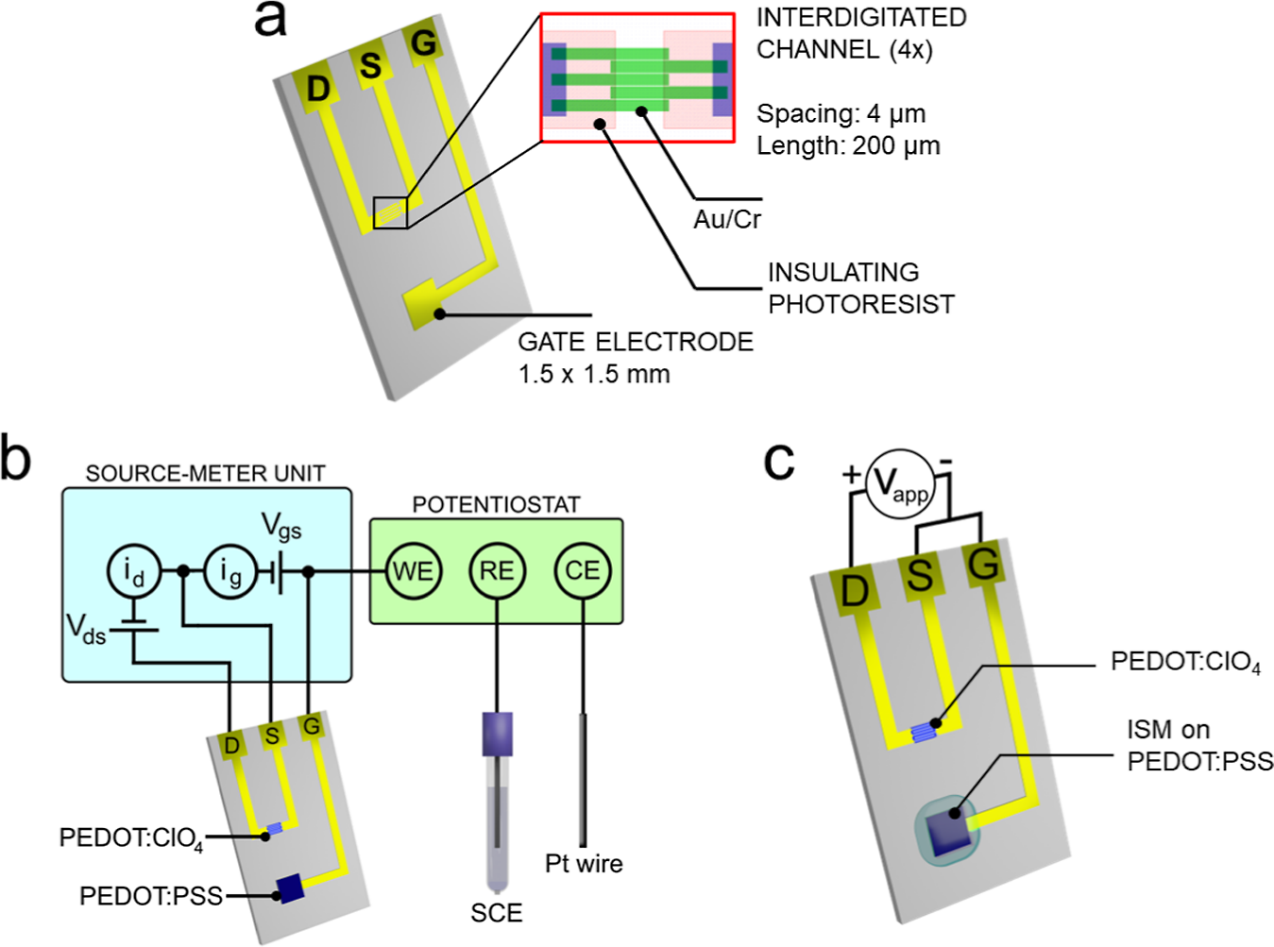
(a) Pseudo-Wrighton OECT geometry. (b) Instrumental setup used for i/E characterizations. (c). Circuit used for the final potentiostatic operation of a pseudo-Wrighton OECT.

### *i*/*V*, *i*/*E*, and Morphological Characterizations

Using a device not functionalized with the membrane in a classic OECT configuration, composed of PEDOT:ClO_4_, while immersed in 0.1 M KNO_3_, both on the channel and the gate, transfer curves were obtained by imposing a fixed drain–source (*V*_ds_) voltage bias equal to +0.1 V while linearly sweeping the gate-source voltage (*V*_gs_) from 0.0 to 1.0 V at a speed of 10 mV/s. A different transfer curve was plotted using the same parameters on an OECT instrument fitted with the ion-selective membrane on the gate electrode in 19 mM CaCl_2_. Output curves were instead produced by sweeping *V*_ds_ from 0 to 0.7 V at a speed of 50 mV/s and stepping the *V*_gs_ from 0 to 0.8 at 0.2 V intervals between each output curve. During each characterization, the drain (*i*_d_) and gate (*i*_g_) currents were also recorded. Successively, a separate set of characterizations was performed, including electrochemical potential measurements. Using the source-meter unit, *V*_gs_ and *V*_ds_ were supplied, while *E*_g_ was measured against a SCE using the potentiostat. Successively, cyclic voltammetry was performed on the gate electrode using a 3-electrode cell, sweeping *E*_g_ from −0.8 to +0.8 V vs SCE at 50 mV/s. Simultaneously, the source-meter unit was used to supply the channel and measure the current through it upon the application of a +0.1 V bias. Finally, using a fully assembled pseudo-Wrighton device fitted with the ion-selective membrane, a potentiostatic Ca^2+^ determination was carried out in an electrochemical cell in 0.1 M KNO_3_ under a voltage bias of +0.1 V while measuring the gate electrochemical potential. The instrumental setup is schematized in Figure 1b. The morphology of the device was later examined by scanning electron microscopy (SEM) imaging: images were taken using a field effect-SEM (FE-SEM), Sigma 300 VP Gemini 1 (from Zeiss) with an electron high tension of 15 kV and employing secondary electrons.

### Pseudo-Wrighton OECTs Ca^2+^ Potentiostatic Measurements

The pseudo-Wrighton devices were tested by externally short-circuiting the gate and source terminals and applying a voltage bias of +0.1 V between the drain and gate and source terminals (Figure 1c). Once immersed in 20 mL of 0.1 M KNO_3_, known CaCl_2_ additions were performed while the current was measured flowing in the channel. The repeatability of the pseudo-Wrighton devices’ response was evaluated by performing 3 calibrations using a single device, while the reproducibility was assessed by comparing the results obtained from 3 different devices in 0.1 M KNO_3_.

### Pseudo-Wrighton OECTs Selectivity Determination

The selectivity coefficients relative to magnesium and sodium were calculated from calibration plots obtained by spiking the solution with, respectively, 50 mM Mg­(NO_3_)_2_ and 50 mM NaCl, following the fixed interference method^34^ for potentiometric determinations applied to a potentiostatic technique.

### Data Processing

The calibration plots were produced by averaging the last 20% of data points relative to the current– and potential–time steady states relative to each concentration.

## Results and Discussion

### Bulk and Gate Electrode ISE Ca^2+^ Potentiometric Response

The potentiometric response of the Ca^2+^ ISE obtained on bulk Au was evaluated to explore the performance of the ion-selective membrane. Figure 2a,b reports the potentiometric response to calcium addition for a bulk Au electrode without the PEDOT:PSS layer, which underlines the crucial effect that an ion-to-electron transducer has on the membrane, reporting a narrow response range along with a poor slope. Figure 2c,d reports the calibration results for a bulk Au/PEDOT:PSS ISE, highlighting the excellent linearity and response range along with a slope close to the Nernstian theoretical value. Once the viability of the membranes was assessed, other ISEs were produced using microfabricated Cr/Au gate electrodes. The range of calcium concentrations tested was adjusted to better include the average values of biological interest for calcium ions, as previously displayed in Table 1. The response obtained is shown in Figure 2e,f, featuring an average slope of 19.4 mV dec^–1^ was obtained, with a relative standard deviation (RSD) equal to 9%. Despite the good data dispersion, it can be noted that the slope is lower than the one previously obtained on bulk Au, which can be ascribed to the different nature of the substrates used rather than the membrane thickness. This hypothesis was confirmed by profilometric and SEM analyses performed on the ISM obtained on bulk Au and microfabricated Cr/Au devices, which demonstrated the same value for both membranes (approximately 20 μm). Despite the influence of the substrate on the performance, the microfabricated Cr/Au ISEs were shown to display high enough potentiometric transduction properties to be employed in the following development of the OECT-based devices.

**Figure 2:**
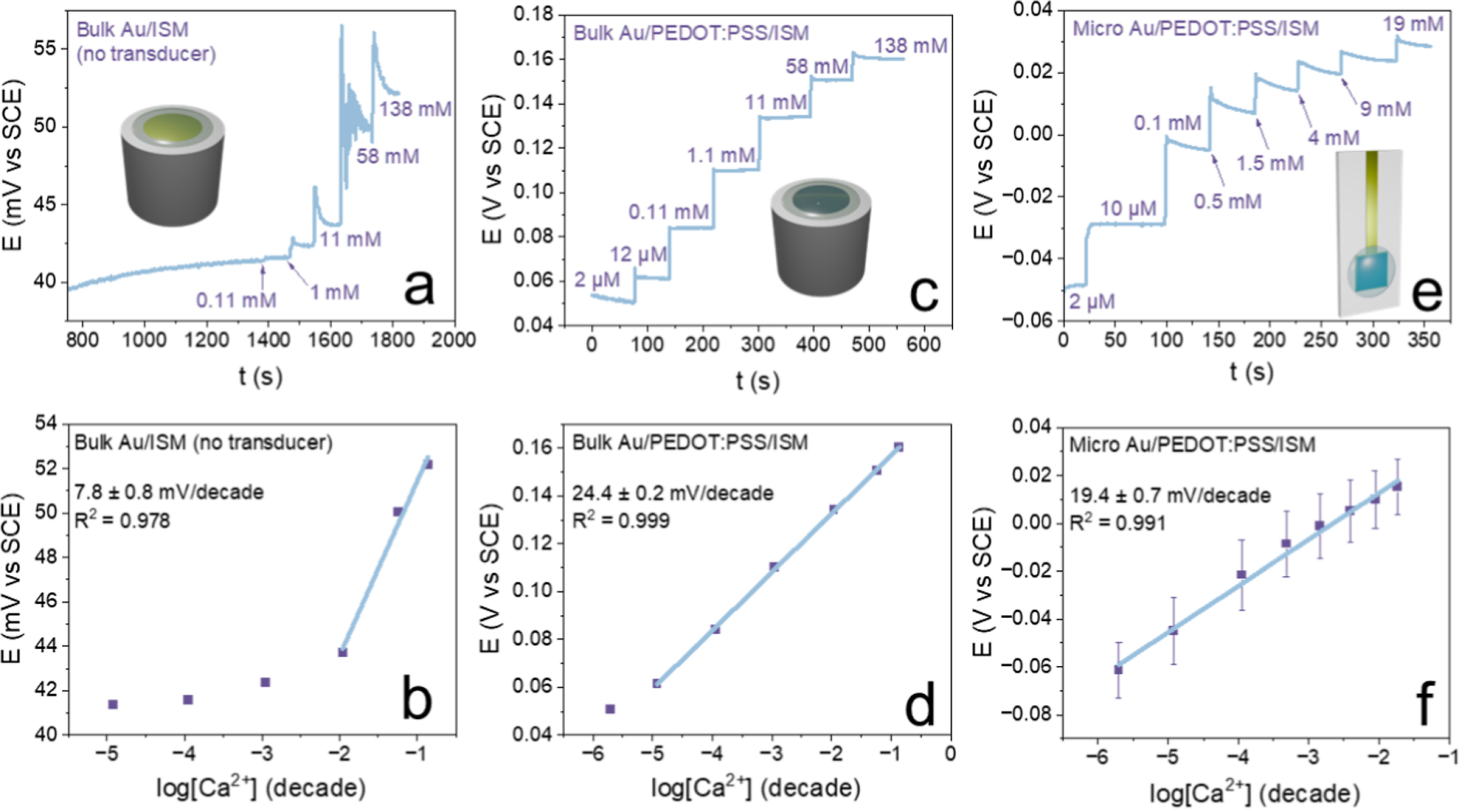
(a) Potentiometric response of a bulk Au calcium ISE and (b) calibration plot. (c) Potentiometric response of a bulk Au/PEDOT:PSS calcium ISE and (d) calibration plot. (e) Potentiometric response of a microfabricated Au/Cr gate electrode/PEDOT:PSS calcium ISE and (f) calibration plot for *N* = 3 repetitions on three separate devices (RSD = 9%).

### Pseudo-Wrighton OECT Development and PEDOT:ClO_4_ Channel Morphological Characterization

After validating the SC-ISE, a study on the most suitable OECT channel material was performed, starting from PEDOT:PSS, as polystyrenesulfonate (PSS) is well known for its cation-exchanging properties, presenting high affinity toward hard-acid cations such as earth-alkali metal.^35^ As a consequence, directly exposing a PEDOT:PSS channel to Ca^2+^ can lead to unwanted changes in the conductivity of the material, as the electron holes on the PEDOT chains are destabilized due to the competitive complexation of Ca^2+^ by the negatively charged PSS units. Specifically, commercial PEDOT:PSS formulations (such as Clevios PH 1000) present a large excess of PSS to PEDOT units, estimated at a 2.5:1 weight ratio,^36^ while PEDOT:PSS films obtained by electropolymerization are usually characterized by a PSS to PEDOT molar ratio lower than one, underlining an excess of PEDOT units,^37^ thus increasing the destabilizing effect of hard cation complexation by PSS on electron holes. To avoid these issues, PEDOT:ClO_4_ was identified as a suitable channel material, as (i) the perchlorate anion is well known for its noncoordinating properties and should not exhibit strong interactions with calcium; (ii) PEDOT:ClO_4_ exchanges anions during redox reactions that lead to electrochemical doping and dedoping in order to maintain the system electroneutrality. Confirmation of this can be found in Figure S2a, where the change in electrochemical potential for a PEDOT:PSS spin-coated and a PEDOT:ClO_4_ electropolymerized film to Ca^2+^ additions is reported, highlighting a stronger response in the former polymer. An additional test involving the effect of known interfering anionic species is reported in Figure S2b, featuring the PEDOT:ClO_4_ response to LiClO_4_ and Na_2_CO_3_. It can be noted that both the ClO_4_^–^ and CO_3_^2–^ anions are capable of shifting the OCP of the material, with the latter ion displaying a stronger effect, due to the double negative charge that favors the formation of a complex with the positive PEDOT:ClO_4_ electron holes. However, considering the negligible concentrations of said species in biological biofluids, such an effect can be considered irrelevant for the purposes of this work. Since PEDOT:ClO_4_ is not readily available as an ink formulation, a different route of preparation was chosen to form the channel, identifying electropolymerization as the most suitable one, necessarily requiring an interdigitated electrode due to the dimensional scale at which the electrosynthesis is effective in producing polymeric channels with electrical continuity. As a consequence, the electrical connection of the gate electrode, originally located under the channel, as shown in Figure S1 in the Wrighton configuration, was changed in order to leave the interdigitated channel unperturbed. For these reasons, the final geometry of the device was based on an interdigitated channel and an adjacent gate electrode (Figure 1a), which allows independent functionalization for each component of the OECT while enabling external short-circuiting of the gate electrode to the source terminal for calcium detection (Figure 1c). This type of sensing strategy finds validation in the previous literature for OECT-based ion sensing,^30,38–40^ where it was demonstrated that Ag/Ag_*n*_X, iridium oxide, and PEDOT:bromothymol blue composites can be used in Wrighton-inspired configurations (i.e., involving different types of gate-channel short circuiting) to exploit variations in X^*n*–^ concentration or pH to drive potentiometric-based changes in channel current. In this work, the potentiometric shift is obtained by functionalizing the gate to obtain a solid-contact Ca^2+^-selective electrode, where the underlying layer of PEDOT:PSS acts as the ion-to-electron transducer, which enables the conversion of the ionic current (constituted by the flow of calcium ions through the ion-selective membrane) into an electric current. Regarding the channel, as previously described, the electrochemical polymerization of PEDOT:ClO_4_ on an interdigitated electrode was chosen as the most viable pathway to not interfere with the purely potentiometric gate action. As such, multiple parameters must be accounted for in order to obtain reproducible and functional OECT channels: the geometry plays a critical role, as the spacing between the electrode fingers needs to be properly selected in order to attain optimal polymer growth and overlapping, usually in the 1–10 μm range, as well as considering the effect of the electric field, which is a relevant factor in determining the morphology of the polymer via nucleation/growth processes.^41–43^ The PEDOT:ClO_4_ interdigitated channel was produced by cyclic voltammetry, according to the 3-step protocol reported in the Experimental Section, whose response is reported in Figure 3a, where a progressive increase in both capacitance and peak current is observed, indicative of polymer film growth, displaying very high reproducibility as reported in Figure S3, where the final electropolymerization cycles for 3 different channels are compared. Optical images of the interdigitated electrodes before and after electrodeposition are reported in Figure 3b, corroborating the hypothesis of a correct electropolymerization of PEDOT:ClO_4_. To better characterize the channel morphology, SEM images were taken, as shown in Figure 3c,d. An irregular globular film structure with an estimated thickness of 6 μm is clearly visible on the Au fingers of the interdigitated channel, caused by the presence of the perchlorate counterion, which, together with electrochemical deposition, favors the formation of well-defined crystalline aggregates,^43–46^ contrary to polystyrenesulfonate.^39–42^ Additionally, it can be noted that there is a slight abundance of material at the edge of every Au finger, with lower amounts of material in the interfinger space, as the higher electric field and boosted EDOT diffusion at the edge favor nucleation of PEDOT domains.^42^ Electrical resistance values of the electrodeposited channels resulted in the order of 100 Ω, thus finally confirming their integrity and usability and proving that PEDOT:ClO_4_ is a viable alternative material to PEDOT:PSS to produce the OECT channels.

**Figure 3:**
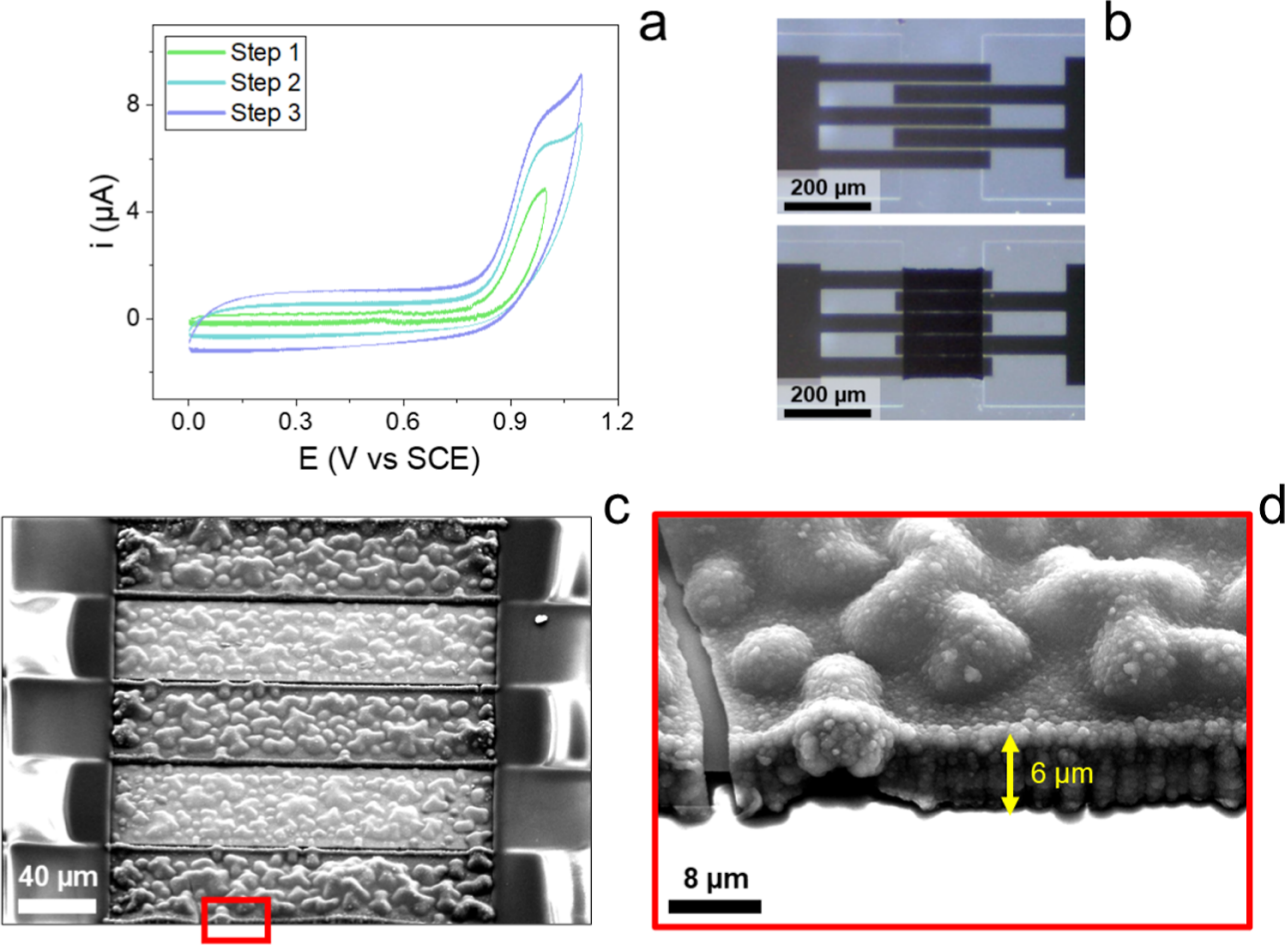
(a) Electropolymerization by cyclic voltammetry of PEDOT:ClO_4_ on the channel. (b) Digital optical microscopy images of the interdigitated channel before and after the functionalization. (c) SEM imaging of a PEDOT:ClO_4_ interdigitated channel, featuring a (d) zoom on the bottom finger.

### Pseudo-Wrighton OECT Electrochemical Characterization

Once the morphological characterization of the channel was completed, the pseudo-Wrighton OECT, featuring a PEDOT:ClO_4_ channel and a pristine PEDOT:PSS gate, was subjected to an investigation of its electrochemical properties. Using the setup described in Figure 1b, a conductivity curve was acquired (Figure 4a) using a source-meter unit to supply the channel with *V*_ds_ = +0.1 V and a potentiostat in a 3-electrode cell setup (RE: SCE; CE: Pt wire), wherein the working electrode terminal is connected to the gate electrode of the transistor configuration. The experimental setup forces the electrochemical potential at the gate electrode (*E*_g_) to simulate the potential variation that is generated by the ion-selective membrane in a calcium sensing experiment, sweeping it from −0.8 V to 0.8 V. The gate electrode was connected to the source meter to observe channel modulation upon gate action. Figure 4a shows that *I*_ds_ increases with increasing *E*_g_. When *E*_g_ is increased, both *E*_s_ (potential of the source terminal measured with respect to a reference electrode) and *E*_d_ (potential of drain terminal measured with respect to a reference electrode) increase as well, and the anodic potential applied to PEDOT:ClO_4_ leads to the PEDOT oxidation (eq 2), highlighting that in comparison to PEDOT:PSS, PEDOT:ClO_4_ interacts electrochemically via the exchange of anions rather than cations, given the absence of a negatively charged ionomer in the matrix capable of storing positively charged ions

2 $$PEDOT+{ClO}_{4}^{-}\to {PEDO\mathrm{T:}ClO}_{4}+e^{-}$$

**Figure 4:**
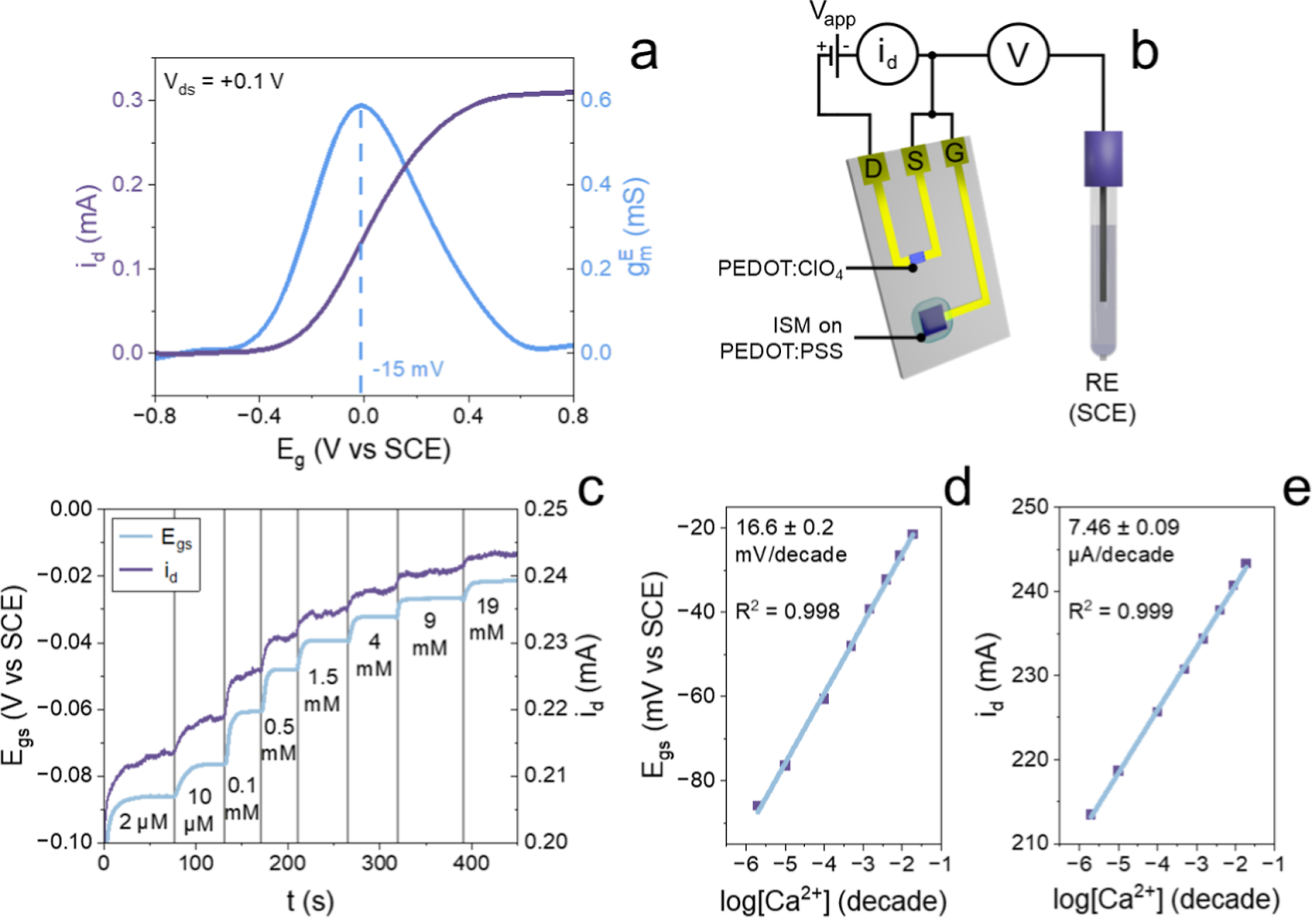
(a) Channel conductivity curve and electrochemical transconductance *g*_m_^E^ plot. (b) Instrumental setup used during the pseudo-Wrighton OECT E/i/V characterization. (c) *E*/*i*/*V* Ca^2+^ sensing under a voltage bias of +0.1 V. Calibration plots obtained using (d) potential and (e) current-based data.

Since holes are injected into the semiconducting polymer, *I*_ds_ increases. The material overall reports an on/off current ratio in the range of 1 × 10^3^, with a threshold electrochemical potential close to −0.2 V vs SCE. Figure 4a proves that the current flowing in the PEDOT:ClO_4_ channel can be effectively changed by the action of the gate electrochemical potential “*E*_g_”, which is the actual parameter driving the shift of the channel conductivity. It can be noticed that apparently the device is turning on for positive *E*_g_ values, due to the difference referencing of the gate^47^ introduced by the coupling of a potentiostat 3-electrode cell system (as described in Figure 1b). The behavior of the transistor overall agrees with our previous work^48^ and the observations of Wrighton’s group, which studied a transistor^41^ with a polypyrrole channel in direct contact with the gate electrode. Therefore, the gate electrode directly controls the electrochemical potential *E* of the channel and consequently the doping state of the polymer. Overall, PEDOT:ClO_4_ proved to be a viable material for the development of an OECT channel, highlighting, at the same time, the possibility of using the material to perform electrochemical potential estimates by a simple measurement of its conductivity. In addition, the complete characterization and terminal electrochemical potentials of an all-PEDOT:ClO_4_ OECT are reported in Figure S4, where the p-type depletion behavior was confirmed (akin to PEDOT:PSS) and a maximum transconductance (*g*_m,max_) of 0.5 mS was obtained, indicative of the amplifying properties of the material.

### Pseudo-Wrighton OECT Ca^2+^ Sensing Mechanism

Once the morphological and electrochemical characterization was performed, a fully assembled pseudo-Wrighton OECT was tested for Ca^2+^ detection in 0.1 M KNO_3_ while measuring the gate electrochemical potential under a voltage bias of +0.1 V across the channel. The instrumental setup, exclusively used to study the sensing mechanism and not part of the final setup, is reported in Figure 1b, while the analysis response is available in Figure 4a, showing the *i*/*E*_g_ conductivity curve and the transconductance calculated using the electrochemical potential (*g*_m_^E^). Upon calcium additions, the electrochemical potential of the gate electrode increases along with the drain current, proving that the conductivity of the PEDOT:ClO_4_ constituting the channel is potentiometrically controlled by the solid-contact Ca^2+^ ISE developed on the gate electrode, as previously demonstrated through the construction of the conductivity curve of the material. Moreover, it can be observed that during operation, the gate electrochemical potential assumes values very close to the maximum *g*_m_^E^, highlighting that the device spontaneously operates under the most optimal conditions. A hypothesized step-by-step discretization of the transduction mechanism is reported in Figure S5, stressing that due to the direct electrical contact between the gate and source terminal, equipotentiality must always be maintained. Therefore, when the gate electrode is subjected to an increase in electrochemical potential, electron holes will flow between the gate and the channel until a new equilibrium in E is reached. Until such equilibrium is reached, a transient current will flow through the gate electrode, meaning that the global charge moving through the membrane at the gate electrode is responsible for the change in channel conductivity, akin to what is described in the coulometric method developed by Johan Bobacka.^49^ From the E/i versus time response, the calibration plots relative to the potentiometric and potentiostatic response can be calculated, which are shown in Figure 4d,e. By comparing the results, it can be noticed that both possess a very high correlation coefficient and the same range of linear response, stretching from 2 μM to 19 mM, which is the same range that was obtained when using the gate electrode as a standalone SC-ISE. It is worth pointing out that the lower limit of linear response could be further extended to lower concentrations since 2 μM is the calcium concentration present as an impurity in the 0.1 M KNO_3_ support electrolyte, as reported by the supplier. Regarding the slope for each calibration, a comparison cannot be made through normalization since the transduction mechanism involved is different between the two extracted parameters, the former relying on electrochemical potential measured against a reference electrode, while the latter relying on drain current recorded by a source-meter unit. Therefore, an alternative method is needed to efficiently compare the sensing performances while neutralizing the influence of the detection system. In such cases, a parameter known as analytical sensitivity can be used, which is a dimensionless value calculated as the ratio between the sensitivity of a device and its associated error.^50^ Interestingly, by comparing the E vs SCE and drain current data, the analytical sensitivity was found to be the same for both calibration plots, being equal to 83, despite the drain current signal being subjected to higher noise. In that regard, the average signal-to-noise ratio obtained for the E-based plot was 1100 (calculated as the ratio between the steady-state signal for every Ca^2+^ concentration and its corresponding standard deviation), while the i-based plot reported a value of 925, underlining the quality of the current-based signal. This is a consequence of the transduction mechanism involved in the pseudo-Wrighton OECTs, which is entirely ruled on a potentiometric basis and therefore capable of fully transmitting the potential-based signal variations to the current-based one. Since both measurements possess the same properties, it can be stated that the pseudo-Wrighton OECTs developed here are equivalent in sensing performance to a standard potentiometric detection system. The advantage of this novel category of devices lies in the absence of a reference electrode needed to perform measurements and in the ease of miniaturization of the sensor. In addition, being a 2-terminal device, the associated readout electronics can also be dramatically simplified, contributing to lowering the cost for the production and use of these novel sensors. Moreover, since the device is based on the use of well-established PVC ion-selective membranes, it presents a very high degree of customizability, as the membrane can be properly tailored for the selective detection of any ion by using its appropriate ionophore. Finally, repeatability and reproducibility trials were carried out in the same conditions in 0.1 M KNO_3_, reporting a relative standard deviation of 14% and 20% obtained by comparing the response of 3 different devices, respectively, as summarized in Figure S6. While being above average for on-field applications, it is worth noting that the device is presented as a proof-of-concept, requiring further optimization in terms of automation of the production process, with a particular focus on the gate functionalization.

### Pseudo-Wrighton OECT Selectivity

In the development of PoC devices for diagnostic applications, selectivity represents a crucial factor in determining the reliability of the sensor and the range of applications for which it can be used. When ion sensing is focused on, the main concern is represented by other ions present in the sampling environment that might pose interference issues. For this reason, quantitative data regarding the selectivity coefficients for sodium and magnesium ions were gathered according to the fixed interference method,^34^ which relies on the Nikolsky–Eisenman equation; that for a single interfering ion present in solution is described as in eq 3

3 $$E=k+\frac{RT}{nF}\ln\left[a_{A}+K_{A,B}^{pot}a_{B}^{\left(z_{A}/z_{B}\right)}\right]$$

where *E* is the measured potential; “*k*” is a constant that includes the electrode constant, the reference electrode potential, and the junction potential; *z*_A_ and *z*_B_ are charge numbers of the primary ion “A” and of the interfering ion “B”; *a*_A_ and *a*_B_ are the activities of the primary ion “A” and the interfering ion, B; and *K*_A,B_^pot^ is the potentiometric selectivity coefficient for the primary ion “A” against the interfering ion “B”. *R*, *T*, and *F* are, respectively, the universal gas constant, temperature in *K*, and Faraday’s constant. The common procedure involves performing a calibration using analyte ion “A” in the presence of a fixed concentration of interfering ion “B”. The intersection between the lower plateau and the linear response range (effectively equivalent to the Limit of Detection calculated by graphical means) is used to calculate the activity of primary ion “A”. Then, by knowing the activity of the interfering ion “B”, it is possible to calculate the selectivity coefficient using eq 4

4 $$K_{A,B}^{pot}=\frac{a_{A}}{a_{B}^{\left(z_{A}/z_{B}\right)}}$$

It must be noted that the fixed interference method by the Nikolsky–Eisenman equation is commonly used for standalone ISE, whereas in our case, the final signal output by the device is potentiostatic (i.e., current-based). However, considering that the signal transduction is potentiometric and performed by an ion-selective membrane, thus representing a Nernstian system, this method was considered as transposable from pure potentiometric systems to potentiostatic analogues, approximating the activity of the ions of interest with their concentration. Two calcium calibrations were performed using the same device, first in a 0.1 M KNO_3_ solution containing 50 mM NaCl and then in a 0.1 M KNO_3_ solution containing 50 mM Mg­(NO_3_)_2_. The response and calibration plots are reported in Figure 5, highlighting the intersection point used to calculate “*a*_A_”, approximated with the concentration of Ca^2+^, while the data obtained for the 2 sets of lines are reported in Table 2. Through this method, we obtained coefficients equal to 0.003 for sodium and 0.001 for magnesium. Interestingly, sodium ions presented a higher interfering action on the device, hence the higher selectivity coefficient value. This result could be ascribed to similarity in size rather than charge since sodium has an ionic radius^51^ of 102 pm, which is close to the calcium one, equal to 100 pm. However, the interfering action is overall limited by the fact that sodium is a monovalent ion with a soft acid character (according to the hard and soft acid and bases theory), which leads to lower interactions with the calcium ionophore, which features oxamide groups with a hard-base character. In addition, the lipophilicity of such cations can be used to explain the results: by comparing the charge/radius ratio (expressed in *e* pm^–1^, where “*e*” is the elementary charge) for the interfering species, respectively, 0.028 and 0.010 for Mg^2+^ and Na^+^, it is possible to estimate the hydration energy of the cations and thus their hydrophilicity. In this case, it clearly emerges that in relative terms, sodium will present a higher lipophilicity than magnesium, diffusing more easily in the plasticized PVC membrane.

**Figure 5:**
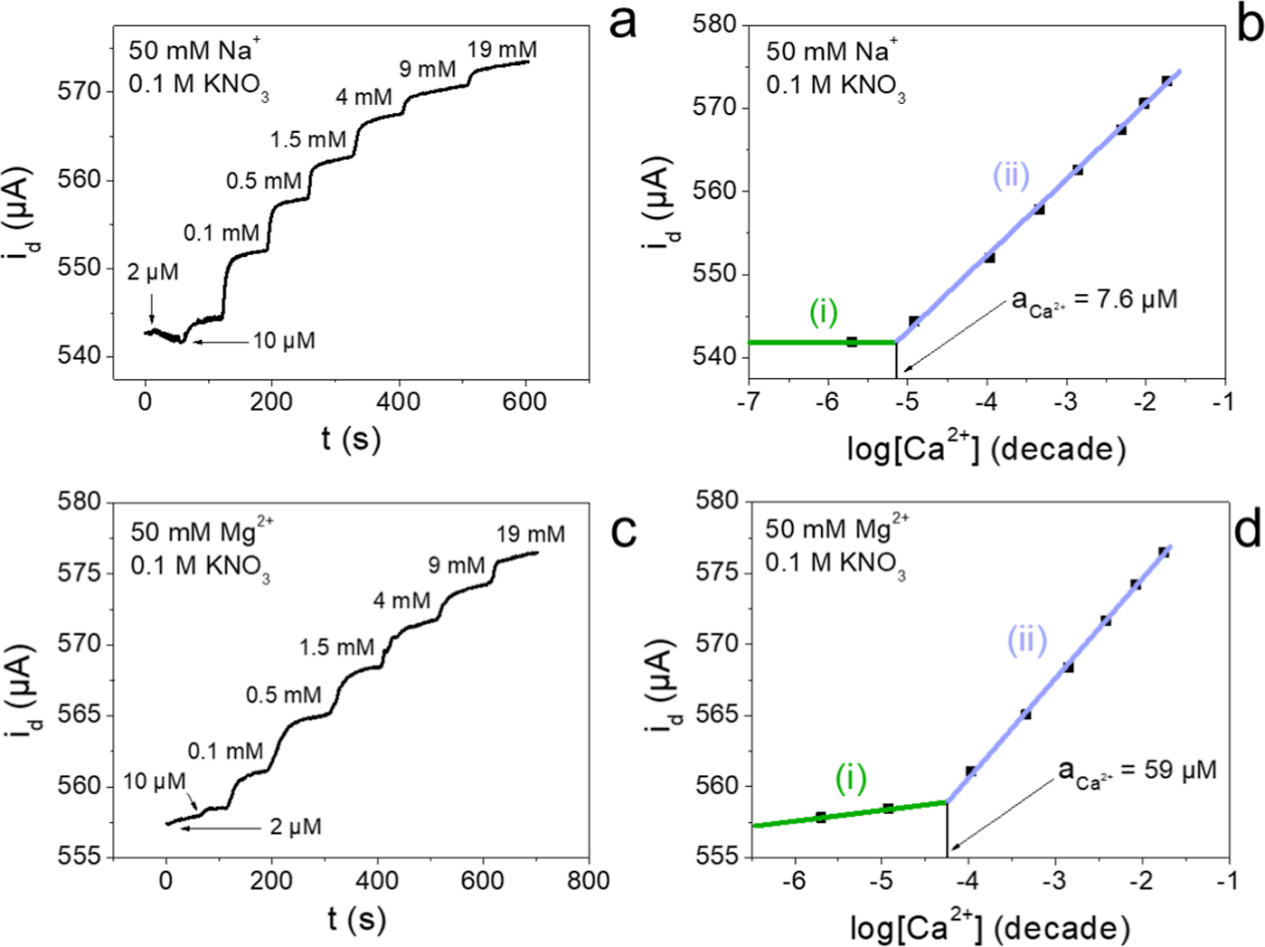
Potentiostatic response for the fixed interference method for (a,b) sodium and (c,d) magnesium during potentiostatic calcium sensing in 0.1 M KNO_3_ (*V*_app_ = +0.1 V).

**Table 2:** Linear Regression Data for the Fixed Interference Method Tests for Sodium and Magnesium in Potentiostatic Calcium Determination in 0.1 M KNO_3_ and Calculated Selectivity Coefficients for *V*_app_ = +0.1 V

	50 mM Na^+^ (Figure 5b)		50 mM Mg^2+^ (Figure 5d)	
	slope (μA dec^–1^)	intercept (μA)	slope (μA dec^–1^)	intercept (μA)
(ii)	9.145	588.7	6.997	588.6
(i)	0	541.9	0.812	526.2
*a*_Ca^2+^_	7.6 μM	56 μM		
*K*_A,B_^pot^	0.003	0.001		

After determination of the selectivity coefficients, qualitative data regarding the interference posed by some of the main components of wound exudate were obtained. Moreover, despite not being present in appreciable amounts in the human body, an additional interference test was performed to better investigate the selectivity of the ion-selective membrane. Figure 6a features the response of a pseudo-Wrighton OECT to glucose, sodium lactate, urea, and magnesium ions in comparison to calcium. The interferent concentrations were chosen based on the average value reported in wound exudate,^12^ resulting in no significant change. Moreover, Figure 6b displays the results of the selectivity test performed by using metal cations, mainly focusing on divalent cations. Among the compounds tested, only strontium was shown to cause a detectable interference in the output signal. Specifically, a 10 mM addition of Sr^2+^ caused a current variation of 0.1%, while the same addition of Ca^2+^ induced a 0.9% increase. This effect is mainly related to ionic radius similarity,^52^ as calcium and strontium are, respectively, 100 and 118 pm in radius. Instead, barium and magnesium feature ionic radii too different from calcium to produce any noticeable interference, being, respectively, 135 and 72 pm. Despite the response given by the strontium ion, it must be noted that its biological concentration is negligible compared to calcium, averaging 0.13 μM in whole blood.^53^ To further assess the robustness of our sensor and its reliability in complex media, the pseudo-Wrighton OECT was tested in synthetic wound exudate (SWE), a complex model matrix that emulates the wound environment. The sensor response and calibration curve are presented in Figure 6c,d. The same response was obtained, characterized by increased signal stability that can be attributed to the high solution viscosity caused by the high protein load (22 g/L albumin), which dampens the diffusion of the analyte in the solution, resulting in fewer spikes produced during the addition. Similarly, a good calibration was reported (*R* ^2^ = 0.991) with a slightly narrower linear response range between 10 μM and 19 mM, attributed to calcium complexation by the phosphate ions present in the SWE, still useful for calcium quantification in wound exudate. Moreover, the same slope was achieved when comparing the results previously obtained in 0.1 M KNO_3_, being equal to 7.4 ± 0.3 μA dec^–1^. Overall, the devices proved to be fully capable of reliably responding in synthetic wound exudate in the biological range of interest for calcium, displaying at the same time high selectivity toward organic and inorganic interferents.

**Figure 6:**
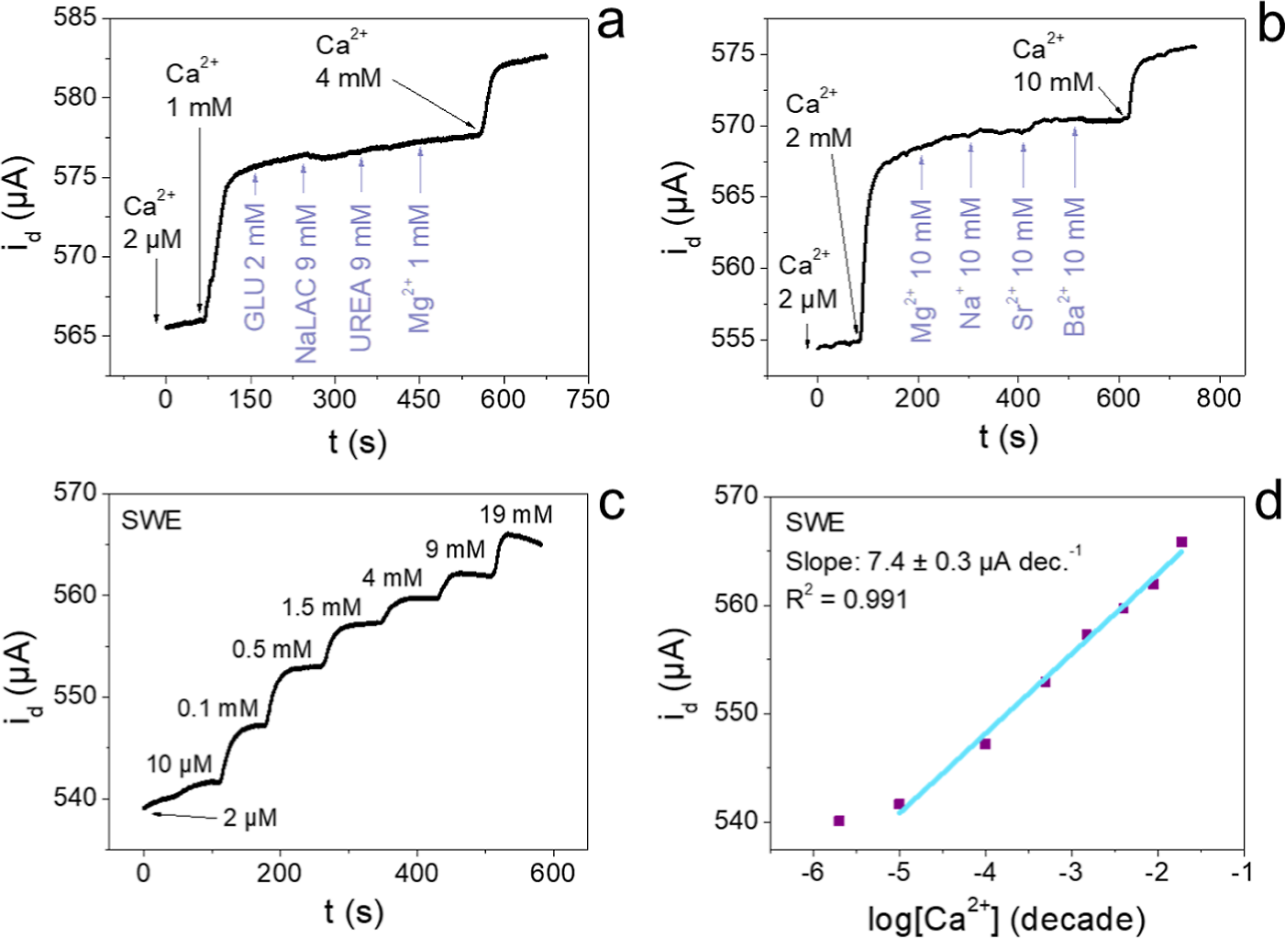
Selectivity tests performed in 0.1 M KNO_3_ using (a) compounds in the biological range for wound exudate and (b) metal cations. (c) Potentiostatic response for Ca^2+^ sensing in SWE and (d) relative calibration curve. All analyses performed under a voltage bias of +0.1 V.

### Comparison with State-of-the-Art OECT Sensors for Ca^2+^ Detection

Given the medical relevance of calcium ions, a vast number of devices can be found in recent literature concerning their detection, with the most popular transduction being based on pure potentiometric systems featuring ion-selective membranes. However, alternative derivations^54^ of apparatuses involving membranes have found applications in transistor-based sensors for calcium ion quantification, which are reported in Table 3.

**Table 3:**
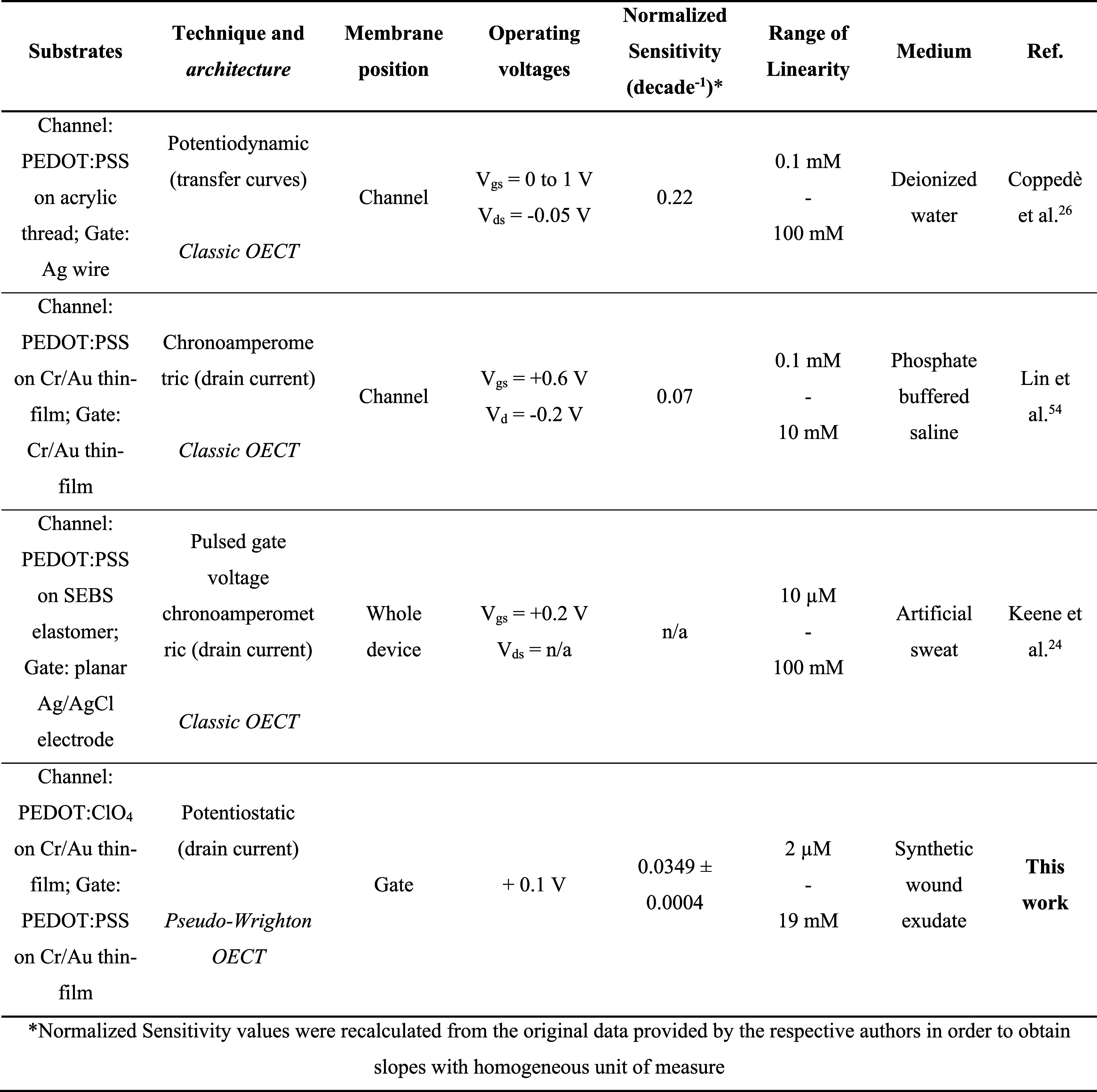
Pseudo-Wrighton OECT Properties’ Comparison with the State of the Art for OECT-Based Ca^2+^ Sensing

For calcium ion sensing, the literature is dominated by classic OECT systems, highlighting the novelty of our pseudo-Wrighton-based OECT configuration. All of the OECTs reported in the above table rely on a substantially different transduction mechanism, which could benefit from additional electrochemical characterizations. Generally speaking, PEDOT:PSS channels are covered with the ISM, allowing the selective access of calcium, acting as the only cation capable of interacting with the channel during the doping–dedoping reactions and thus as the chemical species responsible for the changes in the output signal. When the normalized sensitivity is compared, the values of the OECT-based sensors are higher than those of the pseudo-Wrighton sensor. In this regard, it is worth noting that, thanks to the novel architecture and the careful selection of the transistor materials, the response of our device is exclusively driven by the potentiometric response of the ISM, which is selective to Ca^2+^. Contrarily, devices equipped with Ag/AgCl gate electrodes (especially when not adequately encapsulated, not operated at saturation, or lacking a double junction) are likely to be affected by chloride ion variations in a non-negligible way, which is worth keeping in mind when carrying out a calibration upon additions of Cl^–^ salts. Ag/AgCl electrodes are, in fact, second species indicator electrodes, whose electrochemical potential varies with the Cl^–^ activity in the sample solution by 59 mV dec^–1^. This aspect should be considered in terms of drift and interference produced on the analytical signal during Ca^2+^ sensing in the presence of Ag/AgCl-based electrodes. Nonetheless, the pseudo-Wrighton sensor only requires the application of one small voltage instead of the 2 biases required to operate conventional OECTs. In terms of interference, our devices were tested extensively and quantitatively, displaying excellent selectivity against the most common chemical species found in wound exudate (including synthetic wound exudate) as well as the most biologically relevant ions, leading to the first-time calculation of Nikolsky–Eisenman selectivity coefficients through the adaptation of a potentiometric method in a potentiostatic system. For comparison, the state-of-the-art sensors mainly performed selectivity studies in human sweat while also focusing on the specific effect of biologically common ions such as potassium and sodium. The results reported good selectivity, albeit in a qualitative way, also highlighting an increase in background noise and a shift in the response range. Besides the high selectivity and robustness given by the purely potentiometric transduction and the lower power consumption, the pseudo-Wrighton sensor also exhibits the lowest detection limit, which was associated with the lowest value of the linear response range, as it represents the lowest amount of calcium detectable due to Ca^2+^ impurities presence in the KNO_3_ electrolyte.

## Conclusion

In the field of ion sensing for PoC applications, the most common sensing strategy involves standard potentiometric systems exploiting pseudoreference electrodes, akin to other OECT-based platforms where Ag/AgX composites are often used as gate electrodes. Consequently, such systems are exposed to unwanted potential shifts due to unforeseen chemical changes in the biological environment while at the same time representing an undesired source of Ag^+^ ions. Moreover, the transduction mechanism of existing OECT-based ion sensors, as well as the role of the ion-selective membrane, has not been fully clarified yet and can benefit from additional clarifications and alternative configurations development. In this work, a novel sensor was developed by taking inspiration from Wrighton-style OECTs. An in-depth study was carried out to identify an architecture that would allow the signal to be transduced through a purely potentiometric mechanism generated by the ion-selective membrane. In particular, the OECT channel was fabricated in PEDOT:ClO_4_ to avoid unwanted interactions between calcium and PSS and the device does not contain silver in contact with the electrolyte solution to avoid chloride interference. To the best of our knowledge, this is one of the few examples of an OECT where channel modulation through direct potentiometric action generated by an ion-selective membrane has been clearly demonstrated, measured, and characterized. Specifically, the device is equipped with a silver-free solid-contact ion-selective gate electrode directly shorted to the source, providing an alternative OECT configuration which requires a single voltage bias to operate, offering significant benefits in terms of fabrication, cost, complexity, and readout electronics. Moreover, since ion-selective membranes can be tailored to be selective to a plethora of cations and anions, more pseudo-Wrighton-based sensors selective for different ions can be developed using the same architecture, simply involving the change of the membrane ionophore species. The performances reported by such devices were found to be comparable with the state-of-the-art OECT-based Ca^2+^ sensors, especially in terms of limit of detection, albeit not reporting the highest sensitivity due to the passive, thermodynamically spontaneous gate modulation, which in turn dramatically lowers the power consumption of the device, nevertheless providing a robust sensor for calcium detection in its biological range of interest, without the need for Ag/AgX composites. The careful choice and study of the materials employed enables excellent selectivity against the most common biological interferents as well as high ionic strength solutions, also corroborated by the first-ever calculation of selectivity coefficients for a potentiostatic-operating OECT. In addition, the device proved fully functional in synthetic wound exudate, thus validating its response in complex matrixes, which while not reporting above average performances represent an interesting prototype serving as a basis for future diagnostic PoC applications in wound care. The meticulous study, carried out to achieve a pure potentiometric transduction, provides a promising architecture for selective OECT sensors based on ion-selective membranes, paving the way to the rapid development of new devices for different ions.

## Supplementary Material



## Notes

Structure of a Wrighton-style OECT; PEDOT:PSS and PEDOT:ClO_4_ potentiometric response to calcium ions; reproducibility of PEDOT:ClO_4_ electrodeposition; additional PEDOT:ClO_4_-based OECT electrochemical characterizations; description of the proposed sensing mechanism; and repeatability and reproducibility trials (PDF)

## References

[1] Kumbhojkar S. V., Kale A. D., Kumbhojkar V. R., Desai K. M. (2019). Salivary calcium
as a diagnostic tool for screening of osteoporosis in postmenopausal
women. J. Oral Maxillofac. Pathol..

[2] Patel R. M., Varma S., Suragimath G., Zope S. (2016). Estimation and comparison
of salivary calcium, phosphorous, alkaline phosphatase and pH levels
in periodontal health and disease: A cross-sectional biochemical study. J. Clin. Diagn. Res..

[3] Jayasena C. N., Mahmud M., Palazzo F., Donaldson M., Meeran K., Dhillo W. S. (2011). Utility of the urine calcium-to-creatinine
ratio to diagnose primary hyperparathyroidism in asymptomatic hypercalcaemic
patients with vitamin D deficiency. Ann. Clin.
Biochem..

[4] Heller H. J. (1999). The role
of calcium in the prevention of kidney stones. J. Am. Coll. Nutr..

[5] Subramaniam T., Fauzi M. B., Lokanathan Y., Law J. X. (2021). The role of calcium
in wound healing. Int. J. Mol. Sci..

[6] Lansdown A. B. G., Path F. (2002). Calcium: a potential central regulator in wound healing
in the skin. Wound Repair Regen..

[7] Prompt C. A., Quinton P. M. (1978). Functions of calcium
in sweat secretion. Nature.

[8] Metzler-Wilson K., Wilson T. E. (2016). Impact of calcium regulation on eccrine sweating and
sweating disorders: The view from cells to glands to intact human
skin. Exp. Physiol..

[9] Foley K. F., Boccuzzi L. (2010). Urine calcium: Laboratory measurement and clinical
utility. Lab. Med..

[10] Rehak N. N., Cecco S. A., Csako G. (2000). Biochemical composition
and electrolyte
balance of ‘unstimulated’ whole human saliva. Clin. Chem. Lab. Med..

[11] Sato K. (1977). The physiology,
pharmacology, and biochemistry of the eccrine sweat gland. Rev. Physiol. Biochem. Pharmacol..

[12] Trengove N., Beilefeldt-Ohmann H., Stacey M. (1996). Cytokine profile of wound fluid from
nonhealing and healing chronic leg ulcers. Wound
Repair Regen..

[13] Bobacka J., Bobacka J., Ivaska A., Lewenstam A. (1994). Mechanism
of ionic and redox sensitivity of p-type conducting polymers. J. Electroanal. Chem..

[14] Bobacka J. (2006). Conducting
polymer-based solid-state ion-selective electrodes. Electroanalysis.

[15] Lan W. J., Zou X. U., Hamedi M. M., Hu J., Parolo C., Maxwell E. J., Bühlmann P., Whitesides G. M. (2014). Paper-based
potentiometric ion sensing. Anal. Chem..

[16] Nyein H. Y. Y., Gao W., Shahpar Z., Emaminejad S., Challa S., Chen K., Fahad H. M., Tai L. C., Ota H., Davis R. W., Javey A. (2016). A Wearable Electrochemical Platform
for Noninvasive Simultaneous Monitoring of Ca2+ and pH. ACS Nano.

[17] Jiang C., Yao Y., Cai Y., Ping J. (2019). All-solid-state potentiometric sensor
using single-walled carbon nanohorns as transducer. Sens. Actuators, B.

[18] Kim T., Yi Q., Hoang E., Esfandyarpour R. (2021). A 3D Printed Wearable Bioelectronic
Patch for Multi-Sensing and In Situ Sweat Electrolyte Monitoring. Adv. Mater. Technol..

[19] Pereira A. N., Thomas F., Curry E. T., Butler J., Johnson A., Hossain N. I., Noushin T., Tabassum S. (2022). Flexible Sensor Suite
Integrated into Textile for Calcium Ion and Fall Detection. IEEE Sens Lett..

[20] Seaton B. T., Hill D. F., Cowen S. L., Heien M. L. (2020). Mitigating the Effects
of Electrode Biofouling-Induced Impedance for Improved Long-Term Electrochemical
Measurements in Vivo. Anal. Chem..

[21] Klimov, A. I. ; Zherebin, P. M. ; Gusev, A. A. ; Kudrinskiy, A. A. ; Krutyakov, Y. A. The silver ions contribution into the cytotoxic activity of silver and silver halides nanoparticles. IOP Conference Series: Materials Science and Engineering; Institute of Physics Publishing, 2015; Vol. 98, p 012034.

[22] Smith J. N., Thomas D. G., Jolley H., Kodali V. K., Littke M. H., Munusamy P., Baer D. R., Gaffrey M. J., Thrall B. D., Teeguarden J. G. (2018). All that is silver is not toxic: Silver ion and particle
kinetics reveals the role of silver ion aging and dosimetry on the
toxicity of silver nanoparticles. Part. Fibre
Toxicol..

[23] Rohde M. M., Snyder C. M., Sloop J., Solst S. R., Donati G. L., Spitz D. R., Furdui C. M., Singh R. (2021). The mechanism of cell
death induced by silver nanoparticles is distinct from silver cations. Part. Fibre Toxicol..

[24] Keene S. T., Fogarty D., Cooke R., Casadevall C. D., Salleo A., Parlak O. (2019). Wearable Organic Electrochemical
Transistor Patch for Multiplexed Sensing of Calcium and Ammonium Ions
from Human Perspiration. Adv. Healthcare Mater..

[25] Pierre A., Doris S. E., Lujan R., Street R. A. (2019). Monolithic Integration
of Ion-Selective Organic Electrochemical Transistors with Thin Film
Transistors on Flexible Substrates. Adv. Mater.
Technol..

[26] Coppedè N., Giannetto M., Villani M., Lucchini V., Battista E., Careri M., Zappettini A. (2020). Ion selective
textile organic electrochemical
transistor for wearable sweat monitoring. Org.
Electron..

[27] Estivill M. C., Yazza A. A., Blondeau P., Andrade F. J. (2024). Ion-selective organic
electrochemical transistors for the determination of potassium in
clinical samples. Sens. Actuators, B.

[28] Han S., Yamamoto S., Polyravas A. G., Malliaras G. G. (2020). Microfabricated
Ion-Selective Transistors with Fast and Super-Nernstian Response. Adv. Mater..

[29] White H. S., Kittlesen G. P., Wrighton M. S. (1984). Chemical derivatization of an array
of three gold microelectrodes with polypyrrole: fabrication of a molecule-based
transistor. J. Am. Chem. Soc..

[30] Gualandi I., Tessarolo M., Mariani F., Tonelli D., Fraboni B., Scavetta E. (2019). Organic Electrochemical Transistors
as Versatile Analytical
Potentiometric Sensors. Front. Bioeng. Biotechnol..

[31] Arcangeli D., Gualandi I., Mariani F., Tessarolo M., Ceccardi F., Decataldo F., Melandri F., Tonelli D., Fraboni B., Scavetta E. (2023). Smart Bandaid Integrated with Fully
Textile OECT for Uric Acid Real-Time Monitoring in Wound Exudate. ACS Sens..

[32] Bobacka J., Mccarrick M., Lewenstam A., Ivaska A. (1994). All solid-state poly­(vinyl
chloride) membrane ion-selective electrodes with poly­(3-octylthiophene)
solid internal contact. Analyst.

[33] Gemene K. L., Bakker E. (2009). Measurement of total calcium by flash chronopotentiometry
at polymer membrane ion-selective electrodes. Anal. Chim. Acta.

[34] Zoski, C. G. Handbook of Electrochemistry; Elsevier, 2007.

[35] Gore S., Rane K. (2023). Studying the Effect
of Cross-Linking and Sulfonation on the Calcium-Binding
Ability of Polystyrene Sulfonate in the Presence of Dodecyl Sulfate. Ind. Eng. Chem. Res..

[36] Kim D. H., Lee D. J., Kim B., Yun C., Kang M. H. (2020). Tailoring
PEDOT:PSS polymer electrode for solution-processed inverted organic
solar cells. Solid-State Electron..

[37] Zotti G., Zecchin S., Schiavon G., Louwet F., Groenendaal L., Crispin X., Osikowicz W., Salaneck W., Fahlman M. (2003). Electrochemical
and XPS studies toward the role of monomeric and polymeric sulfonate
counterions in the synthesis, composition, and properties of poly­(3,4-ethylenedioxythiophene). Macromolecules.

[38] Kim Y., Lim T., Kim C. H., Yeo C. S., Seo K., Kim S. M., Kim J., Park S. Y., Ju S., Yoon M. H. (2018). Organic electrochemical
transistor-based channel dimension-independent single-strand wearable
sweat sensors. NPG Asia Mater..

[39] Mariani F., Gualandi I., Tessarolo M., Fraboni B., Scavetta E. (2018). PEDOT: Dye-Based,
Flexible Organic Electrochemical Transistor for Highly Sensitive pH
Monitoring. ACS Appl. Mater. Interfaces.

[40] Mariani F., Serafini M., Gualandi I., Arcangeli D., Decataldo F., Possanzini L., Tessarolo M., Tonelli D., Fraboni B., Scavetta E. (2021). Advanced Wound Dressing
for Real-Time pH Monitoring. ACS Sens..

[41] Kittlesen G. P., White H. S., Wrighton M. S. (1984). Chemical
derivatization of microelectrode
arrays by oxidation of pyrrole and N-methylpyrrole: fabrication of
molecule-based electronic devices. J. Am. Chem.
Soc..

[42] Musumeci C., Hutchison J. A., Samorì P. (2013). Controlling the morphology of conductive
PEDOT by in situ electropolymerization: From thin films to nanowires
with variable electrical properties. Nanoscale.

[43] Lee J., Chhatre S., Sitarik P., Wu Y., Baugh Q., Martin D. C. (2022). Electrochemical Fabrication and Characterization of
Organic Electrochemical Transistors Using poly­(3,4-ethylenedioxythiophene)
with Various Counterions. ACS Appl. Mater. Interfaces.

[44] Baek S., Green R. A., Poole-Warren L. A. (2014). The biological
and electrical trade-offs
related to the thickness of conducting polymers for neural applications. Acta Biomater..

[45] Vlamidis Y., Lanzi M., Salatelli E., Gualandi I., Fraboni B., Setti L., Tonelli D. (2015). Electrodeposition of PEDOT perchlorate
as an alternative route to PEDOT:PSS for the development of bulk heterojunction
solar cells. J. Solid State Electrochem..

[46] Donahue M. J., Sanchez-Sanchez A., Inal S., Qu J., Owens R. M., Mecerreyes D., Malliaras G. G., Martin D. C. (2020). Tailoring PEDOT
properties for applications in bioelectronics. Mater. Sci. Eng. R Rep..

[47] Salvigni L., Nayak P. D., Koklu A., Arcangeli D., Uribe J., Hama A., Silva R., Hidalgo
Castillo T. C., Griggs S., Marks A., McCulloch I., Inal S. (2024). Reconfiguration of organic electrochemical transistors for high-accuracy
potentiometric sensing. Nat. Commun..

[48] Gualandi I., Tessarolo M., Mariani F., Cramer T., Tonelli D., Scavetta E., Fraboni B. (2018). Nanoparticle gated
semiconducting
polymer for a new generation of electrochemical sensors. Sens. Actuators, B.

[49] Bobacka J. (2024). Perspective
on the coulometric transduction principle for ion-selective electrodes. Sens. Actuators, B.

[50] Skoog, D. A. , Holler, F. J. , Crouch, S. R. Principle of Instrumental Analysis, 10th ed.; Cengage, 2016, p 147.

[51] Shannon R. D. (1976). Revised
effective ionic radii and systematic studies of interatomic distances
in halides and chalcogenides. Acta Crystallogr.,
Sect. A.

[52] Chemistry of the Elements, 2nd ed.; Greenwood, N. N. , Earnshaw, A. , Eds.; Butterworth-Heinemann, 1997; pp 107–138.

[53] Piette M., Desmet B., Dams R. (1994). Determination of strontium in human
whole blood by ICP-AES. Sci. Total Environ..

[54] Lin B., Wang M., Zhao C., Wang S., Chen K., Li X., Long Z., Zhao C., Song X., Yan S., Wang L., Ma W. (2022). Flexible organic integrated electronics
for self-powered multiplexed ocular monitoring. npj Flexible Electron..

