# Supplementary material for: Potentiometric Detection of Calcium Ions Using an Organic Electrochemical Transistor
Source: ACS Omega. 2025 Jul 18;10(29):32202–15. doi: 10.1021/acsomega.5c03720 (PMC12311683; doi:10.1021/acsomega.5c03720)
Supplement: Supplementary file 1 [file ao5c03720_si_001.pdf]

Potentiometric detection of calcium ion using an organic electrochemical transistor

Danilo Arcangeli[a,b], Federica Mariani[b], Isacco Gualandi[b], Manuel Ragnucci[b], Francesco Decataldo[c], Filippo Bonafè[c], Domenica Tonelli[b], Beatrice Fraboni[c], Erika Scavetta[b*]

[a]*Organic Bioelectronics Laboratory, Biological and Environmental Science and Engineering Division (BESE), King Abdullah University of Science and Technology (KAUST), Thuwal 23955-6900, Saudi Arabia*

[b]*Department of Industrial Chemistry "Toso Montanari", University of Bologna, Via Piero Gobetti 85, 40129, Bologna, Italy*

[c]*Department of Physics and Astronomy "Augusto Righi", University of Bologna, Viale Berti Pichat 6/2, Bologna, 40127, Italy*

[*]*Corresponding author*

danilo.arcangeli@kaust.edu.sa

KEYWORDS : OECT, PEDOT:ClO_4, calcium sensing, ion selective, Wrighton, PVC membrane

SUPPORTING INFORMATION

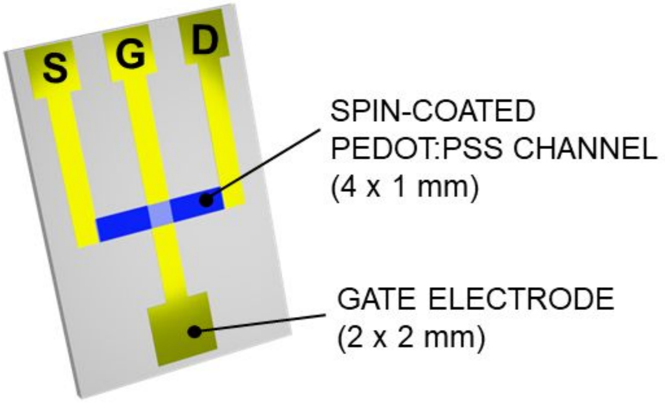

Figure S1 - Wrighton-OECT structure.

PEDOT DERIVATIVES' RESPONSE TO Ca^{2+} ADDITIONS

It is worth noting that as mentioned in the main text, commercial PH1000 presents a higher PSS-to-PEDOT ratio compared to electropolymerized PEDOT:PSS films. Consequently, it can be reasonably expected for the latter material to present an even higher response to calcium, as there are fewer PSS^- units available to stabilize $PEDOT^+$.

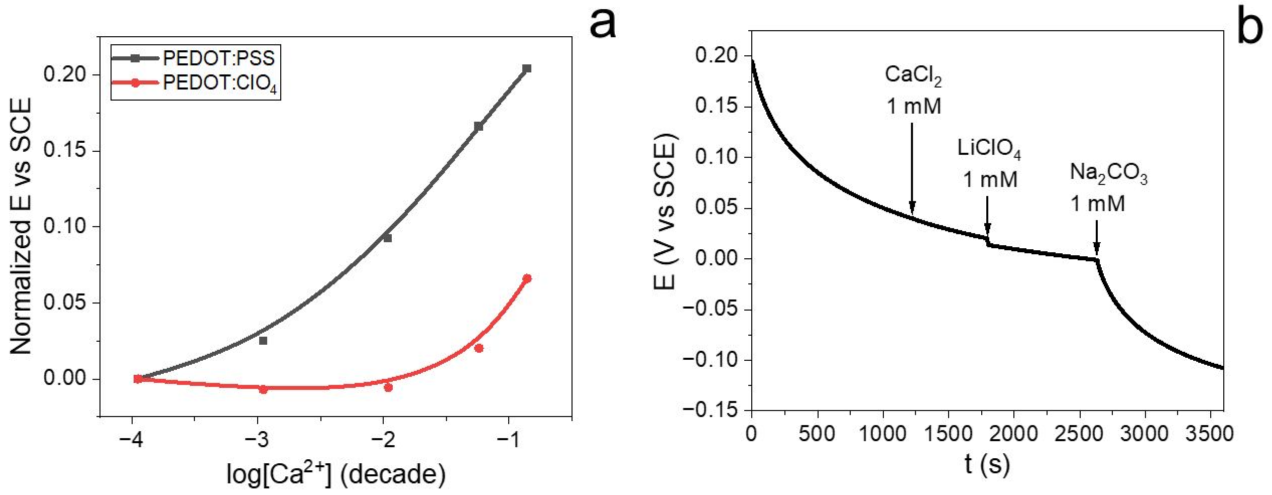

Figure S2 - (**a**) Normalized potential response (at 10 µM $CaCl_2$) to $CaCl_2$ additions for PEDOT:PSS and PEDOT:ClO_4 in 0.1 M KNO_3. PEDOT:PSS was obtained by spin-coating a PH1000-based ink formulation according to the experimental method described in "Wrighton OECTs production" on a microfabricated Cr/Au device. PEDOT:ClO_4 was obtained by cyclic voltammetry on a bulk Au electrode, performing 5 cycles from 0 to 1.1 V vs SCE, at 100 mV s^{-1} in 10 mM EDOT and 0.1 M $LiClO_4$. (**b**) PEDOT:ClO_4 response in 0.1 M KNO_3 comparing $CaCl_2$ to $LiClO_4$ and Na_2CO_3.

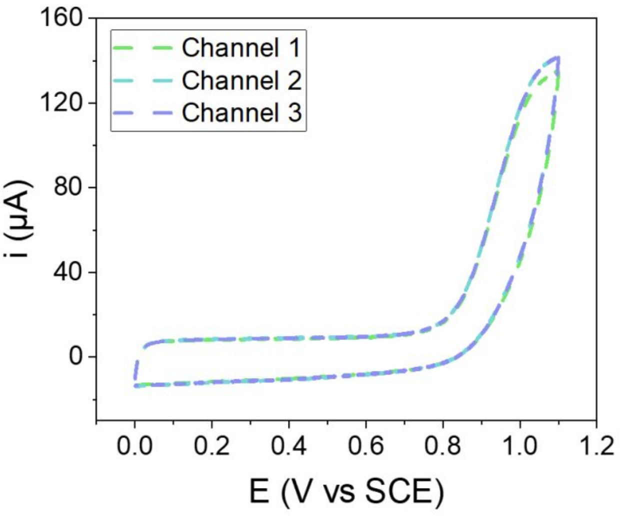

Figure S3 - Comparison of the last cycle for the electrodeposition in cyclic voltammetry of 3 different PEDOT:ClO_4 channels (step 3, 5th cycle, 0 to 1.1 V vs SCE, at 100 mV s^{-1} in 10 mM EDOT and 0.1 M $LiClO_4$).

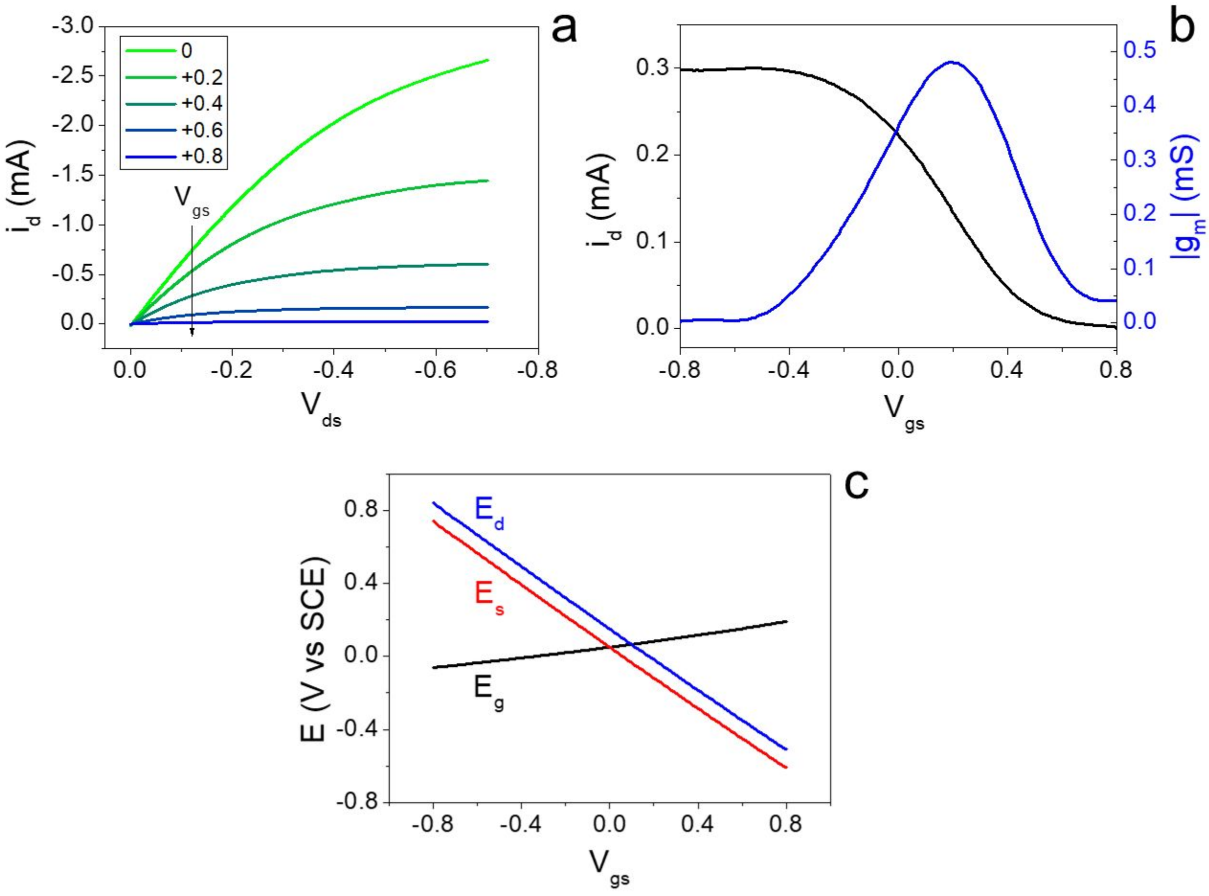

Figure S4 - (**a**) Output and (**b**) transfer-transconductance curves. (**c**) Electrochemical potential measurements performed while scanning the gate-source voltage against a SCE. An all-PEDOT:ClO_4 OECT was used (V_{ds} = +0.1 V).

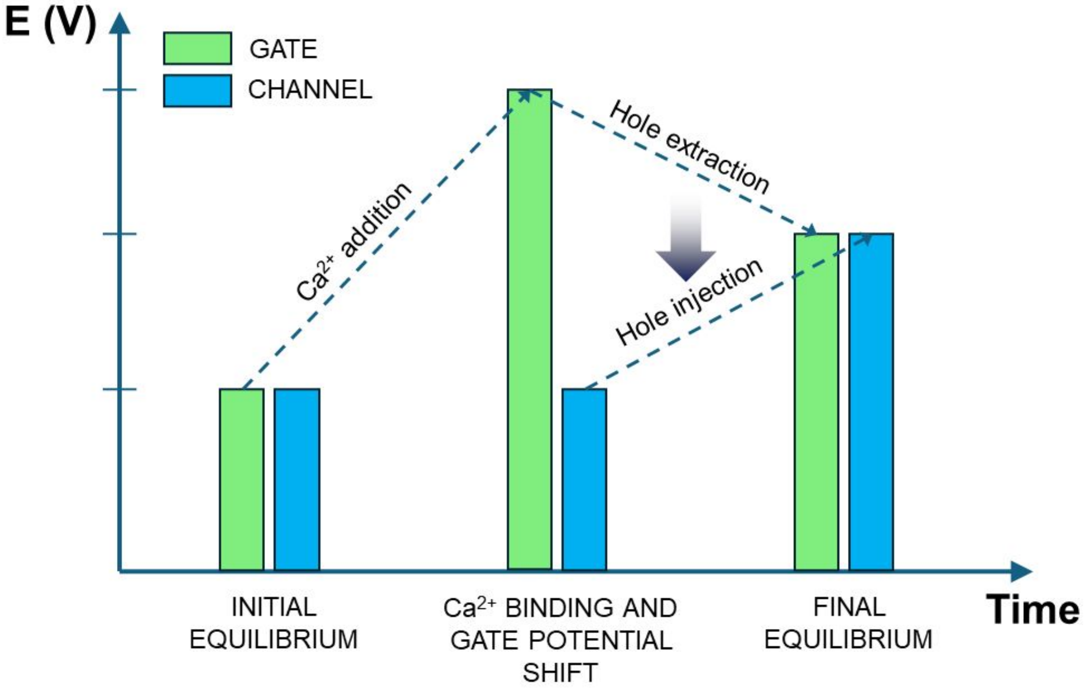

Figure S5 - Hypothesized discretization of the potentiometric transduction mechanism for Ca^{2+} sensing in pseudo-Wrighton OECTs.

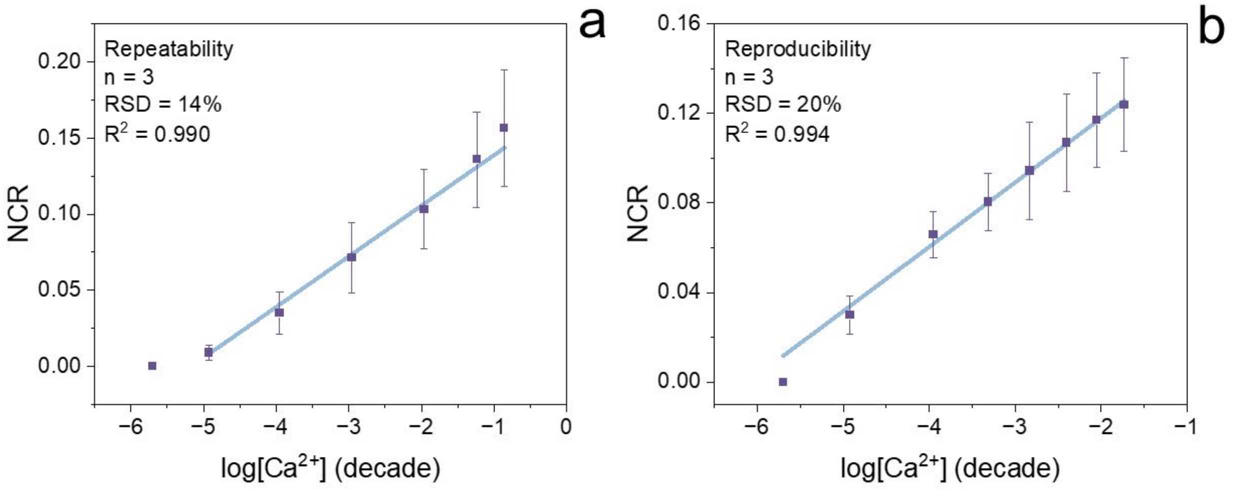

Figure S6 - (**a**) Repeatability and (**b**) reproducibility trials for the pseudo-Wrighton devices performed in 0.1 KNO_3. The current data have been normalized at 2 μM $CaCl_2$.
